# Benefits and Inputs From Lactic Acid Bacteria and Their Bacteriocins as Alternatives to Antibiotic Growth Promoters During Food-Animal Production

**DOI:** 10.3389/fmicb.2019.00057

**Published:** 2019-02-11

**Authors:** Nuria Vieco-Saiz, Yanath Belguesmia, Ruth Raspoet, Eric Auclair, Frédérique Gancel, Isabelle Kempf, Djamel Drider

**Affiliations:** ^1^EA7394-ICV, Institut Charles Viollette, Université de Lille, Villeneuve-d’Ascq, France; ^2^Phileo Lesaffre Animal Care, Marcq-en-Barœul, France; ^3^Laboratoire de Ploufragan-Plouzané-Niort, Agence Nationale de Sécurité Sanitaire de l’Alimentation, de l’Environnement et du Travail (ANSES), Ploufragan, France; ^4^Université Bretagne Loire, Rennes, France

**Keywords:** probiotics, animal health, lactic acid bacteria, antibiotic alternatives, microbiota, immunity

## Abstract

Resistance to antibiotics is escalating and threatening humans and animals worldwide. Different countries have legislated or promoted the ban of antibiotics as growth promoters in livestock and aquaculture to reduce this phenomenon. Therefore, to improve animal growth and reproduction performance and to control multiple bacterial infections, there is a potential to use probiotics as non-antibiotic growth promoters. Lactic acid bacteria (LAB) offer various advantages as potential probiotics and can be considered as alternatives to antibiotics during food-animal production. LAB are safe microorganisms with abilities to produce different inhibitory compounds such as bacteriocins, organic acids as lactic acid, hydrogen peroxide, diacetyl, and carbon dioxide. LAB can inhibit harmful microorganisms with their arsenal, or through competitive exclusion mechanism based on competition for binding sites and nutrients. LAB endowed with specific enzymatic functions (amylase, protease…) can improve nutrients acquisition as well as animal immune system stimulation. This review aimed at underlining the benefits and inputs from LAB as potential alternatives to antibiotics in poultry, pigs, ruminants, and aquaculture production.

## Introduction

Livestock production is one of the fastest growing aspects of the agricultural sector. During the last decades, production, and consumption of animal products have largely increased (Speedy, 2003). This increase will continue in the near future in order to satisfy the high demand for livestock products, such as meat, milk, eggs, and fish, especially in industrialized countries (OECD/FAO, 2018). The dominant livestock types are pig with 112.33 million of tons (MT), poultry (109.02 MT), and cattle, which includes beef and buffalo meat (67.99 MT) representing 91.80% of meat production in the world (FAOSTAT, 2013). While fish captures, and aquaculture production reached 158 MT (FAOSTAT, 2013).

In veterinary medicine, antibiotics are used to fight clinical and subclinical infectious diseases. Notably, in some countries, they are also used as antimicrobial growth promoters (AGPs). To this end, antibiotics are supplied in subtherapeutic doses to provide benefits for livestock by improving growth rate, reducing mortality, and enhancing animal reproductive performance (Marshall and Levy, 2011). Antibiotics mostly used for AGP applications include tetracyclines, ionophores, and penicillins (American Meat Institution, 2013). In Europe, different antimicrobials have been used (Butaye et al., 2003), but the Regulation (EC) No 1831/2003 stated that “Antibiotics, other than coccidiostats or histomonostats, shall not be authorized as feed additives” and these are now banned in the EU. Global antibiotic consumption in livestock was estimated to be approximately 63,000 to over 240,000 metric tons yearly (World Bank, 2017), and these quantities may certainly increase because of the high consumption level registered in the emerging economies (World Bank, 2017). However, a substantial decline of the sales of antimicrobials for food-producing species has been observed in some countries (ESVAC, 2017). As a consequence, this overuse of antibiotics will contribute to spreading antimicrobial resistance worldwide.

Antibiotics can affect the intestinal microbiota and host physiology by (i) preventing pathogen colonization, (ii) impacting the immune system, (iii) increasing fat absorption by decreasing the hydrolysis of conjugated bile salts, and (iv) enhancing the use of nutrients as a result of an alteration of the intestinal wall and lamina propria (Niewold, 2007; Lee et al., 2011). The balance existing between beneficial and non-beneficial bacteria in the gastrointestinal tract (GIT) of a healthy animal can be modified upon alteration of the bacterial proportions causing pathogen infections from different external sources (Kers et al., 2018; Pluske et al., 2018). Pathogenic bacteria can negatively act on the animal health and welfare, as well as on their growth and reproduction performances. Some of these can reach the human gastrointestinal tract through the food chain (Newell et al., 2010), which meanwhile can lead to antibiotic resistance transmission (Soucy et al., 2015). Resistance to antibiotics is a serious concern for humanity. To reduce this phenomenon, the EU ban of antibiotics as growth promoters, should be globalized worldwide. Consequently, some countries such as Mexico, New Zealand, and South Korea have adopted the EU approach. Other countries such as United States, Canada, or Japan have established guidelines and recommendations to reduce the use of AGP in animal productions (Laxminarayan et al., 2015; Brown et al., 2017; Liao and Nyachoti, 2017). To help fighting against antibiotic resistance, the international organizations have ruled through global action plans aimed at ensuring treatment and prevention of infectious diseases with safe and effective medicines (WHO, 2015).

Ban of antibiotics as AGPs is economically and negatively impacting the livestock sector because of different and uncontrolled bacterial diseases (Laxminarayan et al., 2015). To help control increasing resistance to antibiotics, innovative alternatives are urgently needed for food-animal production (Seal et al., 2013; Cheng et al., 2014; Kogut, 2014; Czaplewski et al., 2016). Related to that, the EU has recently recommended the use of alternative strategies in food-producing animals to limit antimicrobial resistance (AMR), and this resulted in temporarily and satisfactory achievements as observed in some EU countries. The strategies adopted included (i) national reduction targets, (ii) benchmarking of antimicrobial use, and (iii) controls on prescription and restrictions on use of specific critically important antimicrobials, together with improvements to animal husbandry and disease prevention and control measures (Murphy et al., 2017). According to studies by Allen et al. (2013), Czaplewski et al. (2016), Seal et al. (2018), additional means expected to replace AGPs in livestock sector are prebiotics, antimicrobial peptides (AMPs), bacteriophages and their gene products, antibodies, vaccines, and natural compounds such as polyphenols and particularly probiotics. The current adopted definition of probiotics from FAO/WHO (2002) states that probiotics are “live microorganisms, providing health benefits for the host, when they are administered in adequate amounts.” Microorganisms with probiotic grade must be devoid of any adverse effects (cytotoxicity, antibiotic resistance, hemolysis), and endowed with beneficial claims. Probiotics are known to act in strain-dependent manner and inhibit pathogenic bacteria through different mechanisms as reported in different studies (Kabir, 2009; Alloui et al., 2013; Pandiyan et al., 2013; Dowarah et al., 2017). Regarding to the functions allocated to probiotics, it has been established that numerous phylogenetic analyses associated to experimental data have revealed some paradigm for host-adaptation (Frese et al., 2011).

According to Krajmalnik-Brown et al. (2012), the animal intestinal microbiota is a key organ, which plays a determinant role in the harvesting, storage, and expenditure of energy obtained from the diet. Notably, these functions can influence the health and weight modification of the animal (Krajmalnik-Brown et al., 2012). Note therefore, that another report from FAO (2013) reported the possibility of probiotics application for animal nutrition as gut ecosystem enhancers. Interestingly, Yirga (2015) and Seal et al. (2018) reported and argued on the use of lactic acid bacteria (LAB)-probiotics in promoting the growth and reproduction performances and the survival rate and health status of animals. Related to LAB-probiotics, Table 1 shows the list of these strains potentially usable as antibiotics replacers because of their multifaceted functions.

**Table 1:** Probiotic genera used in animal farming^∗^.

Animal	Yeast	Bacteria		Fungi	Microalgae
		LAB	Non-LAB		
Poultry	*Candida,* *Saccharomyces* *Kluyveromyces*	*Lactobacillus* *Streptococcus* *Pediococcus* *Enterococcus* *Weissella*	*Bacillus Bifidobacterium*	*Aspergillus*	*–*
Pig	*Saccharomyces Kluyveromyces*	*Lactobacillus Pediococcus Enterococcus* *Weissella*	*Clostridium* *Bacillus Bifidobacterium*	*–*	*–*
Ruminant	*Saccharomyces Trichosporon, Kluyveromyces*	*Lactobacillus Enterococcus*	*Megasphaera* *Bacillus Prevotella Propionibacterium Bifidobacterium*	*Aspergillus*	*–*
Aquaculture	*Saccharomyces Debaryomyces*	*Lactobacillus Lactococcus Leuconostoc Enterococcus Pediococcus Carnobacterium Weissella*	*Bacillus* *Enterobacter Pseudomonas Streptomyces Alteromonas Clostridium Roseobacter Eubacterium Brevibacterium Microbacterium Staphylococcus Streptomyces Micrococcus Psychrobacter*	*Aspergillus*	*Tetraselmis, Phaeodactylum*

*^∗^Data summarized from (Kabir, 2009; Seo et al., 2010; Hai, 2015; FAO, 2016; Banerjee and Ray, 2017; Carnevali et al., 2017; Liao and Nyachoti, 2017).*

**FIGURE 1:**
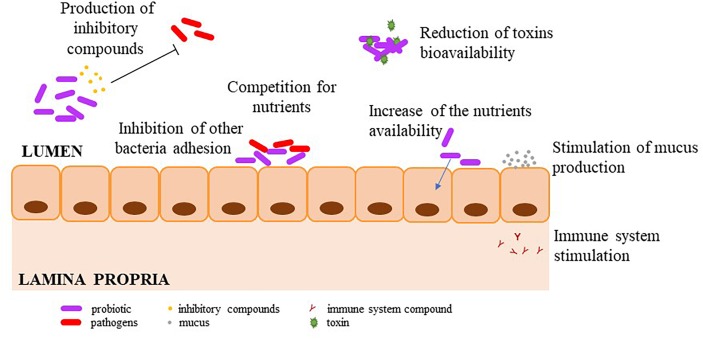
Mechanisms of pathogen inhibition by LAB-probiotics.

Lactic acid bacteria are suitable for livestocks as probiotics because of their capabilities to modify the environment, in which they have been delivered, by producing different metabolites among which a wide range of inhibitory substance and even competitive exclusion (Gaggìa et al., 2010; FAO, 2016). It should be noted that LAB-probiotics belong to *Lactobacillus* (*Lb.*)*, Pediococcus* (*Ped.*)*, Lactococcus* (*Lc.*)*, Enterococcus* (*Ent.*)*, Streptococcus* (*Str*.), and *Leuconostoc* (*Leuc.*) species. Nevertheless, *Lactobacillus* species remain the upmost studied and used ones (Martínez Cruz et al., 2012). Mechanisms of pathogens inhibition by LAB-probiotics include (i) production of inhibitory compounds, (ii) prevention of the pathogens adhesion, (iii) competition for nutrients, (iv) modulation of the host immune system, (v) improvement of nutrient digestibility, feed conversion, and (vi) reduction of toxin bioavailability (Figure 1).

## Pathogen Inhibition

The commonly encountered pathogenic or zoonotic bacteria in food-animal farming are *Escherichia coli*, *Salmonella enterica*, *Campylobacter jejuni*, *Vibrio anguillarum*, *Clostridium perfringens*, *Aeromonas salmonicida*, *Pseudomonas* spp., and *Edwardsiella* spp. (Table 2). Whilst some of these pathogens, such as *V. anguillarum*, and *C. jejuni* are most often encountered in fish and poultry, respectively, other bacteria can affect various hosts provoking different pathologies in several food-producing animals. These are the cases of *E. coli* and *S. enterica* which can afflict poultry, swine, ruminants, and humans (Table 2). As above-cited, LAB-probiotics can limit the dissemination of pathogenic bacteria by mechanisms involving production of inhibitory compounds and competitive exclusion.

**Table 2:** Most frequently encountered bacterial infections among producers in animal production^∗^.

Animal	Potentially reported as pathogenic or zoonotic bacteria
Poultry	*Escherichia coli* *Salmonella enterica* *Clostridium perfringens* *Campylobacter jejuni*
Swine	*Streptococcus suis* *Pasteurella multocida* *Escherichia coli*
Ruminants	*Salmonella abortusovis* *Brucella ovis* *Campylobacter* Enterotoxigenic *Escherichia coli*
Aquaculture	*Aeromonas salmonicida* (Furunculosis) *Vibrio anguillarum* (Vibriosis) *Pseudomonas* spp. *Streptococcus* spp. *Edwardsiella* spp.

*^∗^Data obtained from OIE – World Organization for Animal Health (http://www.oie.int/en/animal-health-in-the-world/oie-listed-diseases-2006/).*

### Production of Inhibitory Compounds

The LAB can produce a wide range of inhibitory compounds to reduce pathogens invasion (Table 3). These include AMPs such as bacteriocins, organic acids, ethanol, diacetyl, carbon dioxide, and hydrogen peroxide (Liao and Nyachoti, 2017).

**Table 3:** Examples of antimicrobial compounds produced by LAB.

Molecule	Examples	Producers	Spectrum	Reference
Bacteriocins	Nisin	*Lc. lactis* subsp. *lactis*	Broad spectrum: Gram-positive bacteria without nisinase	Juncioni de Arauz et al., 2009
	Pediocin PA-1	*Ped. acidilactici*	Broad spectrum: Gram-positive bacteria	Rodríguez et al., 2002
	Enterocin AS48	*Ent. faecalis*	Gram-positive bacteria and *E. coli, Salmonella enterica Bacillus subtilis, B. cereus, B. circulans, Corynebacterium glutamicum, C. bovis, Mycobacterium phlei, Nocardia corallina, Micrococcus luteus, Micrococcus lysodeikticus, S. aureus, Enterococcus faecalis, Ent. faecium, Enterobacter cloacae, E. coli, Klebsiella pneumoniae, Proteus inconstans, Salmonella* Typhimurium*, Shigella sonnei, Pseudomonas fluorescens, P. aeruginosa*	Karpiński and Szkaradkiewicz, 2013; Grande Burgos et al., 2014
	Enterolysin A	*Ent. faecalis*	*Lb. sakei, Lb. brevis, Lb. curvatus, Lc. cremoris, Lb. lactis, Ped. pentosaceus, Ped. acidilactici, Ent. faecium, Ent. faecalis, L. innocua, L. ivanovii, Bacillus subtilis, B. cereus, S. carnosus, Propionibacterium jensenii*	Karpiński and Szkaradkiewicz, 2013
Bacteriocin-like inhibitory substance (BLIS)		*Ped. acidilactici* Kp10 *Leuc. mesenteroides* 406 *Lc. lactis* subsp. lactis CECT-4434	*L. monocytogenes* *L. monocytogenes* *Staphylococcus aureus*	Wong et al., 2017; Arakawa et al., 2016; Souza Vera et al., 2018
Antibiotic	Reutericyclin	*Lb. reuteri*	Gram-positive bacteria (*Lactobacillus, Bacillus, Enterococcus, Staphylococcus*, and *Listeria*)	Rattanachaikunsopon and Phumkhachorn, 2010; Singh, 2018
	Reuterin	*Lb. reuteri* DSM 20016	Gram-positive (*Clostridium* and *Staphylococcus*) and Gram-negative (*Escherichia, Salmonella, Shigella*) bacteria, against the yeast, *Saccharomyces cerevisiae*, and against the protozoan, *Trypanosoma cruzi*	Stevens et al., 2011
Organic acids	Lactic acid, Acetic acid	LAB	Broad spectrum: bacteria affected by pH	
Hydrogen peroxide		*Ped. acidilacti Leuc. mesenteroides Lb. brevis Lb. plantarum* *Lb. casei*	Broad spectrum: Catalase negative bacteria	Whittenbury, 1964
Others	Ethanol	*Bifidobacterium longum Ent. faecalis Lb. acidophilus Lb. fermentum Lb. plantarum* *Weissella confusa*	Broad spectrum: bacteria affected by membrane dissociations	Elshaghabee et al., 2016
	Diacetyl	*Lb. plantarum Lb. helveticus Lb. bulgaricus* *Ent. faecalis* *Leuc. mesenteroides*	*Listeria, Salmonella, Escherichia coli, Yersinia*, and *Aeromonas.*	Singh, 2018
	Carbon dioxide	Heterofermentative LAB	Broad spectrum: Aerobic bacteria	Singh, 2018

Bacteriocins are ribosomally synthesized AMPs produced by both Gram-negative and Gram-positive bacteria (Drider and Rebuffat, 2011). Bacteriocins produced by LAB referred here as LAB-bacteriocins are most often devoid of cytotoxic traits (Belguesmia et al., 2010), and endowed with antagonistic functions as well as additional beneficial attributes (Drider et al., 2016; Chikindas et al., 2018). LAB-bacteriocins are emerging as a novel wave of antibiotics with potent *in vitro* and *in vivo* activities (Stern et al., 2008; Rihakova et al., 2010; Al Atya et al., 2016; Jiang et al., 2016; Caly et al., 2017; Seddik et al., 2017). In contrast to traditional antibiotics, LAB-bacteriocins target specific species and do not affect other population within the same ecosystem. LAB-bacteriocins are known to exert either bacteriostatic or bactericidal activity toward sensitive organisms. Their modes of action have been widely but not thoroughly investigated. Recent insights on modes of action are reviewed elsewhere (Cavera et al., 2015; Drider et al., 2016; Woraprayote et al., 2016; Ben Lagha et al., 2017; Perez et al., 2018). Combinations of LAB-bacteriocins and antibiotics are emerging as novel therapeutic options for food-producing animals (Naghmouchi et al., 2010, 2011, 2013; Al Atya et al., 2016). Different reports have established the main advantages and synergistic actions of LAB-bacteriocins with other biomolecules. These are the case of enterocin AS-48 and ethambutol against *Mycobacterium tuberculosis* (Aguilar-Pérez et al., 2018), nisin and citric acid against *Staphylococcus aureus* and *Listeria monocytogenes* (Zhao et al., 2017), nisin and beta-lactams against *Salmonella enterica* serovar Typhimurium (Rishi et al., 2014; Singh et al., 2014), and Garvicin KA-farnesol against a set of Gram-positive and Gram-negative bacteria (Chi and Holo, 2018). Orally administration of these substances is a challenge because of their enzymatic degradation. This case was reported *in vivo* for lacticin 3147 and nisin (Gardiner et al., 2007; Gough et al., 2018).

Organic acids, including short chain fatty acids, lactic and formic acids, were shown to inhibit potentially pathogenic bacteria of importance for livestock animals. LAB are producing lactic acid as the main product of sugar metabolism (Russo et al., 2017). However, LAB metabolically known as hetero-fermentative species can concomitantly produce other end-products such as acetic acid (Oude Elferink et al., 2001; Schnürer and Magnusson, 2005). Organic acids are known to act by reducing the intracellular pH and inhibiting the active transport of excess internal protons which requires cellular adenosine triphosphate (ATP) consumption leading to cellular energy depletion (Ricke, 2003). The main targets of organic acids are the bacterial cell wall, cytoplasmic membrane, and specific metabolic functions (e.g., replication and protein synthesis) of pathogenic microorganisms leading to their disturbance and death (Surendran Nair et al., 2017; Zhitnitsky et al., 2017). Lactic acid produced by LAB induces an unfavorable local microenvironment for pathogenic bacteria (Dittoe et al., 2018). Wang C. et al. (2015) showed that concentrations of 0.5% (v/v) lactic acid could completely inhibit growth of pathogens such as *Salmonella* spp., *E. coli* or *L. monocytogenes*. Nevertheless, these acids do not affect animal epithelial cells because of the mucus layer that creates a gradient of pH (Allen and Flemström, 2005). Feeding with organic acids such as propionic has some limits because their dissociation before they reach the small intestine (Hume et al., 1993).

Ethanol produced by hetero-fermentative LAB is generated from the glycolysis pathway (Elshaghabee et al., 2016). Ethanol affects the membrane fluidity and integrity resulting in bacterial cell death due to plasma membrane leakage (Ingram, 1989). Oh and Marshall (1993) reported that ethanol concentration at 5% inhibited *L. monocytogenes* replication.

Diacetyl is produced from citrate uptake and metabolism in LAB. Notably *Lb. plantarum, Lb. helveticus, Lb. bulgaricus, Ent. faecalis*, and mainly *Leuc. mesenteroides* and *Lc. lactis* biovar diacetylactis are the most commonly known LAB species producing diacetyl (García-Quintáns et al., 2008; Singh, 2018). Diacetyl interferes with arginine utilization by reacting with the arginine-binding protein of Gram-negative bacteria (Lindgren and Dobrogosz, 1990), while carbon dioxide liberated in the near environment by LAB creates an anaerobic environment where aerobic bacteria cannot grow (Singh, 2018).

Some species of LAB are able to produce hydrogen peroxide (H_2_O_2_) and can inhibit pathogenic bacteria devoid of catalase (Mitchell et al., 2015), through superoxide anion chain reaction enhancing toxic oxidation. H_2_O_2_ bactericidal action depends on the concentrations and environmental factors such as pH and temperature (Surendran Nair et al., 2017). *Lc. lactis* and *Ent. faecium* species were reported to produce H_2_O_2_ with strong antimicrobial effects (Yang et al., 2012).

Reuterin, is a secondary metabolite associated with glycerol metabolism by *Lb. reuteri.* This potent inhibitory compound has a broad spectrum of activity exerted in a pH-independent manner. Reuterin is known to inhibit DNA replication and is resistant to proteolytic and lipolytic enzymes (Singh, 2018). In terms of spectrum of activity, reuterin was shown to be active against *E. coli*, *Staphylococcus aureus*, and *Candida* spp. (Helal et al., 2016).

### Competitive Exclusion

Competitive exclusion (CE) can occur after addition of any culture containing at least one non-pathogenic bacteria to the gastrointestinal tract of animals. This will decrease the number of pathogenic bacteria, through direct or indirect competition for nutrients and adhesion for sites in the gut (Callaway et al., 2008). These LAB-probiotics are able to form biofilms and communicate through Quorum Sensing (QS) upon producing and releasing of autoinducers (Tannock et al., 2005). Pathogens are not able to adhere to the intestinal mucosa, which blocks the development of their population by the constant flow of digesta (Callaway et al., 2008; Yirga, 2015; Liao and Nyachoti, 2017). Some studies aimed at highlighting this mechanism are briefly described below. Carter et al. (2017) reported the protective effect exerted by the combination of 5 × 10^7^ CFU/mL of *Lb. salivarius* 59 and *Ent. faecium* PXN33 by reducing *Salmonella* Enteritidis S1400 colonization in chicks. Penha Filho et al. (2015) reported the effect of 1 × 10^5^ CFU of a *Lb.*-based probiotic suspension on reducing *Salmonella* colonization when this strain was provided to chicks from 1 to 7 days of age. Arruda et al. (2016) showed that a commercial probiotic of *Lactobacillus* spp. decreased prevalence of *Clostridium difficile* infections in pigs when administrated at a concentration of 7.5 × 10^9^ CFU. Sha et al. (2016a) showed through feeding trial assays that 1 × 10^9^ CFU/mL of *Lb. pentosus* HC-2 strongly inhibited the growth of *Vibrio parahaemolyticus* in the intestine of shrimp.

### Modulation of the Host Immune System

Lactic acid bacteria have been widely described for their capabilities to enhance the animal immune system, by positively affecting the innate and adaptive immune response (Tellez et al., 2012; Tsai et al., 2012; Ashraf and Shah, 2014) by helping protect from pathogen disorders (Ashraf and Shah, 2014). The innate immune system induces immediate defense against infection, but also activates a long-lasting adaptive immunity. Components of the innate system recognize the molecular patterns associated with pathogens through pattern recognition receptors (PRRs). The recognition of these patterns by PRR leads to the induction of inflammatory responses and innate host defenses. In addition, the detection of microbes by PRR expressed in antigen-presenting cells, particularly dendritic cells (DC), leads to the activation of adaptive immune responses, by means of T and B cells (Iwasaki and Medzhitov, 2015). Various immune cells types, including granulocytes, macrophages, DCs, and T and B lymphocytes, are involved with inflammatory responses which are mediated by several cytokines like TNFα, IL-1β, IL-6, IL-8, and IL-15 interleukins. The anti-inflammatory/suppressive responses are mediated for their part by IL-10, IL-12, and TGFβ (Hardy et al., 2013). LAB have different immunomodulatory properties associated with their capabilities to induce cytokine production, impact immunomodulation by innate or adaptive immune responses (Kiczorowska et al., 2017a). Those immunomodulation abilities are considered as a crucial criterion for probiotic assessment, through various mechanisms (Wells, 2011; Hardy et al., 2013).

#### Enhancing Innate Immune Response

The primary mechanism of innate immunity stands as physical and chemical barriers such as the intestinal epithelial cells (IECs), which prevent pathogens spreading and subsequent infections (Riera Romo et al., 2016). Depending on the presence of changing mucus layer, probiotics will be able to interact with IECs and DCs (Lebeer et al., 2010). IECs are the first defense barriers (Dowarah et al., 2017) and they are supposed to be the first and most important target cells for probiotic action (Lebeer et al., 2008). Probiotic strains have been shown to have beneficial effects related to the nutritional function of the intestinal epithelium (Lebeer et al., 2008). They also improve intestinal barrier function by stimulating: (i) the production of antimicrobial mucus and peptides such as defensins (Yang et al., 1999; Schlee et al., 2008), (ii) increased immune responses, (iii) improved expression and/or localization of tight junction proteins, (iv) preventing apoptosis of epithelial cells, and (v) induction of cytoprotective molecules (Ewaschuk et al., 2008; Anderson et al., 2010; Mathias et al., 2010; Madsen, 2012). In chicks, *Lb. plantarum* LTC-113 (1 × 10^9^ CFU) can up-regulate cellular junctions to impede pathogens colonization as *Salmonella* (Wang et al., 2018).

In addition, other cellular components of the innate immune system such as monocytes and macrophages, prevent the invasion of pathogens by secreting pro-inflammatory cytokines and cytotoxic molecules. Natural Killer (NK) cells produce cytokines such as interferon (IFN-γ) and various interleukins (IL): IL-10, IL-3, etc. (Vivier et al., 2011). In weaning pigs, administration of *Lb. brevis* ZLB004 (2 × 10^9^ CFU/kg of feed) increased the level of IFN-γ (Liu et al., 2015). Lactobacilli strains as *Lb. fermentum, Lb. salivarius, Lb. crispatus*, and *Lb. gasseri* have been reported to modulate positively the secretion level of the pro and anti-inflammatory interleukins IL-6, IL-8, and IL-10 in order to regulate the inflammation and restore the physiological equilibrium of food-producing animals (Pérez-Cano et al., 2010; Luongo et al., 2013; Sun et al., 2013; Rizzo et al., 2015). IL-8 is a pro-inflammatory cytokine which directly recruits macrophages and leukocytes into inflammatory regions by chemosensing (Riera Romo et al., 2016). A *Lb. acidophilus* strain stimulated anti-inflammatory properties in enterotoxigenic *E. coli* (ETEC) infected piglets when administered at 1 × 10^8^ CFU in the feed. This strain was also able to down-regulate pro-inflammatory cytokines IL-8 and TNF-α *in vivo* based on animals experiments (Li et al., 2016). The same trend in IL-8 levels was also observed in *Aeromonas hydrophila* infected carp (*Cyprinus carpio* Huanghe var.) when fed with 1 × 10^7^ CFU g/L *Lb. delbrueckii* strain (Zhang et al., 2017). Conversely the combination at 1 × 10^7^ CFU each/g feed pellet of *Lc. lactis* BFE920 and *Lb. plantarum* FGL0001 induced IL-8 and IL-6 increase in *Streptococcus iniae*-infected Japanese flounder fish (Beck et al., 2015). Walter (2008) and Brisbin et al. (2011) support that discrepancies in the immunomodulation effects were ascribed to the *Lactobacillus* strains selected, their dosage, and also to the animal conditions. Zhang et al. (2017) established that *Lb. delbrueckii* at 1 × 10^7^ CFU g/L increased levels of anti-inflammatory cytokines IL-10 and TGF-β in the intestine and enhanced immunity of *A. hydrophila-*infected *Cyprinus carpio* Huanghe var.

#### Enhancing Adaptive Immune Response

The adaptive immune response depends on B- and T-lymphocytes, which induce antigen-specific response. Association of *Lb. plantarum* (1 × 10^7^ CFU/kg of feed) and *Clostridium butyricum* (1 × 10^6^ CFU/kg of feed) on production performance and immune function in broilers revealed the increase of IgG and IgA levels in chickens fed with these beneficial probiotics (Han et al., 2018). In direct line, *Lb. plantarum* B2984 was shown to stimulate immunoglobulin production (IgM and IgA) against *Salmonella* infection in pigs during orally challenged animal trials (Naqid et al., 2015). Feeding piglets with a strain of *Lb. rhamnosus* ATCC 7469, at a concentration of 10^9^ CFU/mL, prevented acute infectious diarrhea by stimulating the adaptive immune system subsequently to produce an increase in the concentration of lamina propria CD3^+^ CD4^+^ CD8^-^ T cells (Zhu et al., 2014). In chicken, feed supplementation with 1 × 10^9^ CFU/kg of *Lb. acidophilus* LA5 increased the production of CD4^+^, CD8^+^, and TCR1^+^ T cell in GI tract but also in peripheral blood (Asgari et al., 2016). The administration of 10^10^ CFU/mL *Lactobacillus* spp. could efficiently activate the immunity of mucosa in chickens by increasing the levels of IgA and IgG (Rocha et al., 2012). In young calves, administration of 1.85 × 10^7^ CFU/L of *Lactobacillus* species has been shown to improve weight gain and immunocompetence (Al-Saiady, 2010). In summary, LAB were reported to improve the host resistance and production performance as delineated in Section “Production Performance,” by enhancing the immune response (Kiczorowska et al., 2017a).

### Improvement of Nutrient Digestibility and Feed Conversion

Feed digestibility reflects the amount of absorbed feed and the nutrient’s availability used for the animal growth and reproduction (ILCA, 1990). It is possible to measure the apparent or real digestibility by comparing nutrients contained in the feces from nutrients contained in the dietary intake, or adding external markers (such as titanium dioxide, chromium oxide and rare-earth elements) into feed (ILCA, 1990; Safwat et al., 2015). The improvement of this nutrient digestibility is measured by an indirect method: feed conversion ratio, a correlation between weight of feed administered over the lifetime of an animal and weight gained, which is a valuable indicator of feeding systems efficiency (Wenk et al., 1980; Fry et al., 2018). LAB-probiotics improved nutrient digestibility because of their highly fermentative activities. Indeed, they can enhance the whole digestive process, the metabolic utilization of nutrients, and improve the feed efficiency by producing digestive enzymes (e.g., amylases, chitinases, lipases, phytases, proteases) or by just generating volatile fatty acids and B-vitamins: riboflavin, biotin, B12 vitamin (Russo et al., 2014; Liao and Nyachoti, 2017; Sharifuzzaman and Austin, 2017). In addition, LAB-probiotics can indirectly modify the gut microsystem (FAO, 2016) by helping in the assimilation of nutrients through activation of the host immune cells and increasing the number of antibodies leading to animal welfare improvement (Forte et al., 2016).

### Reduction of Toxin Bioavailability

Protective effects of LAB-probiotics can result in inhibition of toxin expression in pathogens (Liao and Nyachoti, 2017). It was also reported that LAB can constitute natural barriers against mycotoxins considered as potentially harmful compounds for animals (Tsai et al., 2012; Peng et al., 2018). Several investigators reported that *Lb. plantarum, Lb. acidophilus, Lb. paracasei*, and *Ent. faecium* could mitigate the effect of aflatoxins for improving human and animal health (Gratz et al., 2007; Ahlberg et al., 2015; Abbès et al., 2016; Damayanti et al., 2017; Li et al., 2017).

Lactic acid bacteria act as a biological barrier in the intestinal tract, decreasing availability of ingested mycotoxins and neutralizing their adverse effects. Detoxifying capabilities of LAB are associated with their capacities to bind and sequester mycotoxins, boosting their excretion by digestive system in the feces (Zoghi et al., 2014; Damayanti et al., 2017; Li et al., 2017). Mycotoxins can bind to viable and non-viable bacterial surface by adhesion to their cell wall components (Damayanti et al., 2017; Li et al., 2017), and aflatoxin B1 (AFB1), for example, was bound to LAB by non-covalent interactions (Haskard et al., 2001). Tests aimed at controlling mycotoxins have been carried out with *Lb. casei* DSM20011, *Lb. casei* Shirota (Liew et al., 2018), *Lb. rhamnosus* strains LGG and LC 705 (El-Nezami et al., 1998, 2002; Nybom et al., 2007), *Lb. acidophilus* 24 (Pizzolitto et al., 2012), and *Lb. plantarum Lb. brevis*, *Lb. sanfranciscensis*, from LOCK collection (Piotrowska, 2014). Piotrowska (2014) suggested that the binding to mycotoxins, particularly to ochratoxin was ascribed to hydrophobic properties of the cell wall, and also by electron donor-acceptor and Lewis acid-base interactions. LAB have additional anti-mycotoxin mechanisms based on a study performed on mice wherein the diet was supplemented with *Lb. plantarum* C88 (1 × 10^10^ CFU/mL). This supplementation weakened oxidative stress induced by aflatoxin AFB1. Likewise, this strain decreased lipid peroxidation in serum and the liver due to hepatic damage caused by AFB1 toxicity (Li et al., 2017). LAB produce exopolysaccharides, which are important compounds capable of inhibiting bacterial toxins as reported by Ruas-Madiedo et al. (2010) for the interactions between *Lb. rhamnosus* GG against *Bacillus cereus.* Further mechanisms including pH decrease and blockage of QS have been reported during co-existence of *C. perfringens* type A and LAB particularly *Lb. acidophilus* CGMCC 11878 and *Lb. fermentum* CGMCC12029 (Guo et al., 2017).

## Benefits for Animals and Food Chain

**FIGURE 2:**
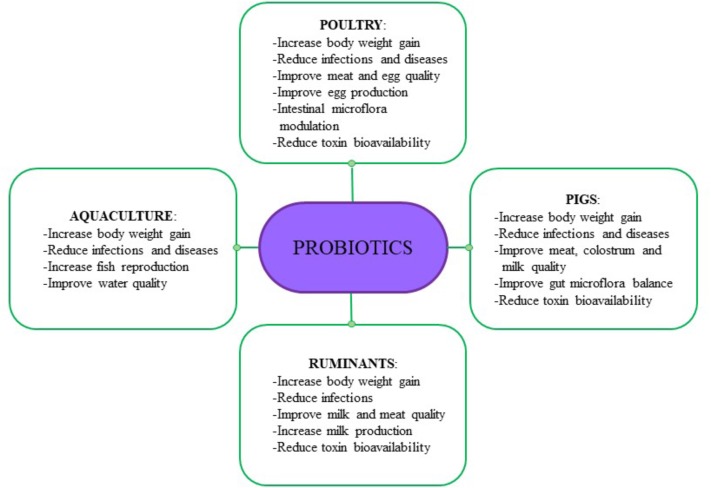
Beneficial effects due to LAB-probiotics administration.

In addition to the above listed positive effects attributed to LAB potentially utilizable as probiotics, we noted further beneficial effects on the health of various livestock animals and quality of animal products. These effects are animal-dependent and consist of enhancing body weight gain, improve gut microbial balance, improve reproductive performance, and increase overall productivity (Figure 2).

### Disease Management

Different investigators have reported the potential effect of LAB to decrease the risk of infections and intestinal disorders associated with pathogens such as *Salmonella* spp.*, E. coli, Campylobacter*, or *Clostridium* spp. LAB-probiotics were used preventively and therapeutically to treat infections by these pathogens (Tellez et al., 2012; Layton et al., 2013; O’Bryan et al., 2014; Gómez et al., 2016; Park et al., 2016).

#### Poultry

Many studies were dedicated to LAB used in poultry. Indeed, Wang et al. (2018) inoculated hatched chicks with 1 × 10^9^ CFU *Lb. plantarum* LTC-113 strain, which provided anti-*Salmonella* Typhimurium protection by limiting the gut colonization and stabilizing the expression of tight junction genes in intestinal epithelial cells among treated chickens making them more resistant to the infection. As previously mentioned, CE is an effective mechanism protecting newly hatched birds from enteropathogen colonization in poultry (Kabir, 2009). Similarly, Olnood et al. (2015) showed that 5 × 10^8^ CFU of *Lb. johnsonii* reduced numbers of *Salmonella enterica* serovar Sofia and *C. perfringens* in challenged 1-day-old Cobb broilers (Olnood et al., 2015). Another study reported that administration of 1 × 10^9^ CFU (50:50) *Lb. salivarius* 59 and *Ent. faecium* PXN33 in combination decreased the levels of colonization in birds by *Salmonella* (Carter et al., 2017). On the other hand, strains of *C. perfringens* are responsible for production of the necrotic enteritis toxins and/or enzymes. Colonization of poultry mucosa by *C. perfringens* occurs during the early life of the birds (M’Sadeq et al., 2015). Several *Lactobacillus* (*Lb. acidophilus, Lb. fermentum, Lb. plantarum, Lb. reuteri*, and *Lb. salivarius*) and *Enterococcus* strains (*Ent. faecium* and *Ent. faecalis*) can inhibit *C. perfringens in vitro* (Caly et al., 2015). The administration of certain LAB strains do not generate systematically benefits for poultry, as underpinned for *Lb. plantarum* PCS 20 against *Campylobacter jejuni* (Santini et al., 2010), or *Lb. johnsonii* FI9785 against *Salmonella* Enteritidis (La Ragione et al., 2004). Therefore, the screening has to be performed in defined conditions and the results are strictly strain-dependent.

Lactic acid bacteria-bacteriocins improve growth performance of *C. perfringens*-challenged chickens allowing weight to recover at similar levels of healthy birds. For instance, this effect was observed when chickens challenged with *C. perfringens* were treated with pediocin A from *Ped. pentosaceus* (Grilli et al., 2009), or when *E. coli* infected chickens were receiving plantaricin from *Lb. plantarum* F1 (Ben Lagha et al., 2017). Notably, anti-*C. perfringens* divercin bacteriocin, produced by *Carnobacterium divergens* AS7 improved growth performance and welfare in treated chickens (Józefiak et al., 2012).

#### Swine

Numerous benefits have been described in pig upon LAB administration specifically with lactobacilli (Yang F. et al., 2015; Valeriano et al., 2016; Dowarah et al., 2017). For pigs, the highest death loss is due to diarrhea caused by ETEC and *Salmonella* (Liao and Nyachoti, 2017). Feed supplementation with various species of *Lactobacillus* including *Lb. johnsonii, Lb. mucosae*, and *Lb. plantarum* improved the gut microbial profile and microbial metabolites production, leading to gut health improvement, and reduced pathogens colonization of intestinal mucosa (Dowarah et al., 2017). Subsequently, these investigators showed that weaned piglets treated with 2 × 10^9^ CFU/g of *Ped. acidilactici* FT28 rather than *Lb. acidophilus* NCDC-15 resulted in the reduction of diarrhea due to dietary and environmental changes (Dowarah et al., 2018). Decrease of ETEC number was observed when a fermented food from reuteran-producer *Lb. reuteri* LTH5794 at a concentration of 1 × 10^7^ CFU/g was administered to weaning pigs. The studied strain was able to produce exopolysaccharides such as reuteran or levan impeding ETEC adhesion to the mucosa (Yang Y. et al., 2015). However, a study from Andersen et al. (2017) showed that no protective effect was observed in newly born piglets treated with commercially available probiotic *Lb. paracasei* F19 strain (2.6 × 10^8^ CFU/kg) and a newly characterized *Ped. pentosaceus* (1.3 × 10^10^ CFU/kg) developed to inhibit *E. coli* F18-inducing diarrhea (Andersen et al., 2017).

*Streptococcus suis* is involved in a wide range of infections in pigs such as meningitis, arthritis, endocarditis, pneumonia, and septicemia (Ben Lagha et al., 2017). This bacterium has been reported to be sensitive to nisin, a well-known and the only bacteriocin marketed now (Lebel et al., 2013).

#### Ruminants

Administration of LAB can prevent development of ruminal acidosis by creating optimal conditions for lactic acid consuming bacteria such as *Megasphaera elsdenii* or *Propionibacterium* spp. (Chaucheyras-Durand and Durand, 2010). Addition of *Lb. acidophilus, Lb. salivarius*, and *Lb. plantarum* at concentrations of 10^7^–10^8^ CFU/g, reduced the incidence of diarrhea in young calves (Signorini et al., 2012). LAB species including *Lb. paracasei* and *Lb. plantarum*, and those isolated from honey like *Lb. kunkeei, Lb. apinorum, Lb. mellis, Lb. mellifer, Lb. apis* had *in vitro* activities against mastitis pathogens such as *Staphylococcus aureus, Staphylococcus epidermidis, Streptococcus uberis*, or *E. coli* (Piccart et al., 2016; Diepers et al., 2017). LAB-probiotics were also successfully used to relieve symptoms of diseases such as coccidiosis, an important parasitic disease of young ruminant livestock, caused by *Eimeria*. These LAB-probiotics minimized the impact of this disease by reducing the risk of dissemination of this parasite (Giannenas et al., 2012). On the other hand, Cao et al. (2007) reported on the effectiveness of intra-mammary infusion of nisin for treating mastitis caused by *Staphylococcus aureus* in dairy cows.

#### Aquaculture

In aquaculture, *Lb.* strains such as *Lb. murinus* or *Lb. pentosus* displayed antagonism against *V. harveyi* and *V. parahaemolyticus*. Other potential LAB-probiotics such as *Ent. faecium* NRW-2 (1 × 10^7^ CFU/g of feed) were reportedly active against *Vibrio* spp. strains through their highly elevated capacities of adhesion and competition for nutrients. These bacteria were reported to decrease the presence of *Vibrio* in shrimp (Sha et al., 2016b). Fish fed with *Lb. delbrueckii* became more resilient to *Aeromonas* infections. It was suggested that 1 × 10^6^ CFU/g of *Lb. delbrueckii* action was linked to their stimulation properties of the shrimp larval gut immune system (Zhang et al., 2017). Araújo et al. (2016) isolated several strains of *Ped. acidilactici* from rainbow trout (*Oncorhynchus mykiss*) feed and larvae. Some of these strains have wide action spectra and showed antagonistic activities against several fish pathogens including *Lc. garvieae, Streptococcus iniae, Carnobacterium maltaromaticum*, and *A. salmonicida*.

Nisin Z-producing *Lc. lactis* WFLU12 provided protection against infections caused by *Streptococcus parauberis* in olive flounder fish (Nguyen et al., 2017). While *Lb. pentosus* 39 produces a bacteriocin active against the fish pathogen *A. hydrophila*, suggesting this LAB could be used as a natural biopreservative agent (Anacarso et al., 2014).

#### LAB-Probiotics in Treatment of Viral Infections

Lactic acid bacteria-probiotics were described as potent organisms to treat viral infections (Al kassaa et al., 2014; Arena et al., 2018a,b). Leyva-Madrigal et al. (2011) reported the capabilities of *Ped. pentosaceus* and *Staphylococcus haemolyticus* (1 × 10^6^ CFU/g feed) to treat white spot syndrome virus (WSSV) in white leg prawns (*Litopenaeus vannamei*). A modified *Lb. plantarum* conferred resistance to yellow head virus in shrimps (Thammasorn et al., 2017). In piglets, *Lb. plantarum* N4 was shown to be active against transmissible Gastroenteritis Virus when provided in a preventive manner (Yang Y. et al., 2017) and *Lb. plantarum* 25F, reduced viral infectivity of Porcine Epidemic Diarrhea Virus (PEDV) (Sirichokchatchawan et al., 2018). The effects of *Lb. casei* CMPUJ 415 and *B. adolescentis* DSM 20083 on rotavirus has been demonstrated by a reduction on the production of a viral enterotoxin protein (NSP4) (Olaya Galán et al., 2016). Roselli et al. (2017) listed numerous examples of LAB-probiotics with antiviral activities such as *Lb. casei* MEP221106 or MEP221114, *Lb. rhamnosus* CRL1505. Nevertheless, Sachsenrödder et al. (2014) hypothesized the administration of probiotics as *Ent. faecium* NCIMB10415 do not have an effect on the virus composition present in the pigs’ gut. For this reason, several studies focus on producing recombinant LAB for vaccination as it is the case of recombinant *Lb. casei* ATCC 393 against PEDV (Wang X. et al., 2017) or *Lb. plantarum* NC8 against H9N2 avian influenza virus (Yang W. et al., 2017) boosting the immune system presenting antigens.

### Production Performance

Lactobacilli produce lactic acid and proteolytic enzymes, which enhance nutrient digestion in the GIT and feed supplementation with *Lactobacillus* strains can induce weight gain in livestock animals (Angelakis, 2017), in a strain strain-specific dependent manner (Drissi et al., 2017). For instance, *Lb. delbrueckii, Lb. acidophilus, Lb. casei, Lb. agilis, Lb. salivarius*, *Lb. fermentum*, and *Lb. ingluviei* were described as weight enhancers (Drissi et al., 2017). The last two species were associated with a substantial weight gain effect in ducks and chicks (Million et al., 2012; Drissi et al., 2017). LAB-probiotics improved the feed conversion efficiency by modifying the intestinal microbiota, limiting the growth of pathogens, promoting non-pathogenic bacteria, and enhancing nutrients use and digestion (Yang Y. et al., 2015). Some LAB-probiotics provided similar benefits as antibiotics in terms of weight gain, feed intake, and feed efficiency, as in the case of *Lb. plantarum* P-8 (2 × 10^6^ CFU/mL) (Wang L. et al., 2015).

Baldwin et al. (2018) showed that a LAB-probiotics consortium containing *Lb. ingluviei, Lb. agilis*, and *Lb. reuteri* strains improved weight gain when they were inoculated early at the hatchery, modifying intestinal microbiota and decreasing the pathogenic taxa numbers in chicks (Baldwin et al., 2018). On the other hand, a feed supplementation with 1 × 10^6^ CFU/g feed *Lb. johnsonii* BS15 in broilers enhanced digestive abilities, promoting growth performance, and lowering body fat content (Wang H. et al., 2017).

Another study carried out on pigs fed with *Lb. plantarum ATCC 4336, Lb. fermentum* DSM 20016, and *Ent. faecium* ATCC 19434 at concentrations of 10^11^ CFU/kg resulted in a weight gain due to the ability of these strains, mainly *Lb.* ones, to produce enzymes enhancing feed digestion, besides lactic acid production (Veizaj-Delia et al., 2010). Lan et al. (2016) showed that a strain of *Lb. acidophilus* increased digestibility in weaning piglets of 14 days-old and improved their growth performance.

However, supplementation of feed with LAB-probiotics did not induce any effect on the animal weight in all cases. Wang et al. (2012) showed that *Lactobacillus* or *Pediococcus* fermented maize feed can modify the microbiota but did not affect pig’s production performances. This variability is related to a heterogeneity between trials. Nonetheless, for pigs, environmental factors such as pen, litter… the sow and piglet are closely related according to Zimmermann et al. (2016).

Concerning ruminants, calves fed with a mixture of 1 × 10^9^ CFU of *Lb. casei* DSPV 318T, *Lb. salivarius* DSPV 315T, and *Ped. acidilactici* DSPV 006T exhibited higher daily weight gain, total feed intake, and starter diet intake (Frizzo et al., 2010). Furthermore, calves fed with 6.8 × 10^8^ CFU of *Lb. acidophilus* and *Lb. plantarum* fortified milk (Gupta et al., 2015) or 1 × 10^10^ CFU of *Lb. plantarum* DSPV 354T (Soto et al., 2014) consumed more milk, in addition to body weight gain and growth performance. A similar effect has been reported for post weaning lambs. When they were fed with Pediococci (1 × 10^6^ CFU/g) an increased intake of dry matter feed and better growth performances were shown (Saleem et al., 2017).

In the aquaculture sector, Zheng et al. (2017) showed a size improvement in white shrimps (*Litopenaeus vannamei*) when their diet was supplemented with 1 × 10^9^ CFU/mL of *Lb. plantarum.* Furthermore, *Lb. delbrueckii* also enhanced the production performance of *Cyprinus carpio* Huanghe var., when added at a concentration of 10^6–7^ CFU/g in feed (Zhang et al., 2017). In abalones (*Haliotis discus hannai* Ino) feed intake and growth increases were observed when *Lb. pentosus* was supplied at concentration of 10^3–5^ CFU/g (Gao et al., 2018).

### Improvement of the Quality of Livestock Products

There are several examples of *Lactobacillus* spp. as probiotic improving meat quality in chickens, including tenderness, appearance, texture, and juiciness among other parameters (Park et al., 2016). It is known that addition of LAB to broiler’s feed reduced the cholesterol content of meat (Popova, 2017). Administration of *Lb. salivarius* SMXD51, at a concentration of 10^7^ CFU/mL, can partially prevent the impact of *Campylobacter* in chickens due to ability of this strain to produce an anti-*Campylobacter* bacteriocin with strong activity against four tested *Campylobacter jejuni* strains (Messaoudi et al., 2012; Saint-Cyr et al., 2017). In addition, the probiotics can improve the production and quality of eggs. Thus, the supplementation of *Ped. acidilactici* MA 18/5M (10^8^ CFU/kg feed) to the chicken feed revealed an effect on the eggs’ quality by increasing their weight, eggshell thickness and decreasing cholesterol on the egg yolk (Mikulski et al., 2012).

In pigs, *Ped. acidilactici* was shown to improve meat quality (Dowarah et al., 2018). Kiczorowska et al. (2017b) compared four groups of cows and demonstrated that cows under an intensive production system receiving a probiotic treatment [*Lb. casei*, *Lb. plantarum* (5 × 10^6^ CFU/mL)], and *S. cerevisiae* (5 × 10^3^ CFU/mL) produced higher milk quality with higher protein content and fat that contained a higher amount of unsaturated fatty acids conversely to cows in intensive production system. It was shown that supplementation of feed with 4 × 10^9^ CFU *Lb. acidophilus* NP51 and *Propionibacterium freudenreichii* NP24 increased milk production and improved apparent digestibility of dietary nutrient (Boyd et al., 2011).

### Fish Reproduction

Probiotics are associated with fish reproduction by enhancing their fecundity rate (Banerjee and Ray, 2017; Carnevali et al., 2017). The direct effects are reportedly due to increasing expression of genes encoding several hormones and enhancing gonadal growth, fecundity, and embryo survival (Gioacchini et al., 2010, 2012). Probiotics also enhance follicules maturation and development, and embryo quality. For example, several strains of *Lb. rhamnosus* can improve the fecundity in zebrafish (*Danio rerio)* model (Hai, 2015; Banerjee and Ray, 2017).

### Water Quality

The quality of water on the fish farms is clearly an important factor to avoid the spread of diseases. The parameters to measure this quality are based on the pH of the water and the amounts of CO_2_, ammonia, nitrate, and phosphorus found in it. *Lb. plantarum* and *Lb. casei* reportedly maintained or improved water quality during fish production (Banerjee and Ray, 2017).

A major concern is related to water pollution with heavy metals provoking fish diseases. This can be avoided by the use of some LAB-probiotics such as *Lb. plantarum* CCFM639, which restored the integrity of damaged tight junction proteins and maintained intestinal permeability leading to decrease of aluminum-induced gut injuries (Yu et al., 2016). Yu et al. (2017) also reported reduction of aluminum accumulation in tilapia tissues and lower death rate upon incorporation of 10^8^ CFU/g *Lb. plantarum* to the feed.

## Synbiotics

Synbiotics are defined as combination of probiotics and prebiotics that beneficially affect the host by improving survival and settlement of live microbial dietary supplements in the gastrointestinal tract. This happens by selectively stimulating the growth and/or by activating the metabolism of one or a limited number of health-promoting bacteria, and thus improving host welfare (Gibson et al., 2004). A prebiotic that confers gastrointestinal health benefits could support the growth of a probiotic which has activity against a potential pathogen (Allen et al., 2013).

Guerra-Ordaz et al. (2014) reported a synergistic effect when *Lb. plantarum* (2 × 10^10^ CFU) and lactulose (10 g/kg feed) were concomitantly used to treat colibacillosis in pigs, reducing diarrhea and improving the average daily weight gain. Yasuda et al. (2007) showed that *Lb. casei* subsp. *casei* (1 × 10^7^ CFU/kg feed) and dextran (5%, w/w) in combination improved the milk yield and milk components in cows, and they hypothesize the symbiotic association had a prophylactic effect inhibiting mastitis development. Data from Naqid et al. (2015) suggested caution on the use of synbiotics because intra-interaction can occur within the combination and reduce the expected activity. Mookiah et al. (2014) showed same body weight increase in chickens treated with multi-strain *Lactobacillus* probiotic at 1 × 10^9^ CFU/g or prebiotic (isomalto-oligosaccharides; 5 or 10 g/kg of feed) separately or their association as synbiotic. Szczurek et al. (2018) showed that synbiotic composed of whey lactose and *Lb. agilis* did not show any advantage when compared to each compound alone.

In fish, the administration of *Ent. faecalis* and mannan-oligosaccharides enhanced growth, immune response, and survival of Rainbow trout (*Oncorhynchus mykiss*) to the infection of *A. salmonicida* (Rodriguez-Estrada et al., 2013). In Tilapia, there is an effect against *A. hydrophila* when the combination of 1 × 10^8^ CFU *Lb. brevis* JCM 1170 or *Lb. plantarum* JCM 1149 and fructo-oligosaccharides (1 g/kg feed) were added to the feed, but the same combination did not improve animal growth or feed conversion (Liu et al., 2017).

## Conclusion and Future Design of Lab-Probiotics

This review highlighted numerous advantages from LAB-probiotics used in animal farming and production. After the indiscriminate use of antibiotics in livestock to increase the animal performance, resistance to these molecules has dramatically escalated. To help reduce this worldwide concern, the use of LAB-probiotics stands as an efficient and promising alternative. Different benefits have been observed in animals fed with various LAB-probiotics. As supported by a variety of studies, LAB-probiotics can control the development of bacterial diseases, increase weight gain in healthy and affected animals, stimulate the quality of the (by-) products of this industry or even improve aquaculture water quality. LAB-probiotics can control bacterial infections by excretion of inhibitory compounds, or by other mechanisms including competitive exclusion, decreasing bioavailability of toxins, strengthening intestinal barrier or positively stimulating the immune system. Their actions are exerted in strain and host-specific manners. Finally, there are a variety of synergistic effects when combining LAB with other probiotic species, prebiotics, or enzymes. In terms of future design, recombinant LAB-probiotics may offer additional advantages. Pioneering studies have already opened this avenue. Indeed, it has been reported that *Lb. plantarum* NC8 can produce a recombinant dendritic cell-targeting peptide with activity against H9N2 avian influenza virus in chickens (Shi et al., 2016; Wang X. et al., 2017). Also, this recombinant dendritic cell-targeting peptide can be used synergistically to enhance vaccine humoral immune responses and to reduce viral replication in chicken lungs (Shi et al., 2016).

## Ethics Statement

All authors of this paper have read and approved the final version submitted. The contents of this manuscript have not been copyrighted or published previously. No procedures performed in these studies have been conducted in human participants and/or animals.

## Author Contributions

All authors listed have made a substantial, direct and intellectual contribution to the work, and approved it for publication.

## Conflict of Interest Statement

The authors declare that the research was conducted in the absence of any commercial or financial relationships that could be construed as a potential conflict of interest. The handling Editor declared a past co-authorship with one of the authors DD.

## References

[B1] Abbès S. Ben Salah-Abbès J. Jebali R. Younes B. R. Oueslati R. (2016). Interaction of aflatoxin B1 and fumonisin B1 in mice causes immunotoxicity and oxidative stress: possible protective role using lactic acid bacteria. *J. Immunotoxicol.* 13 46–54. 10.3109/1547691X.2014.997905 25585958

[B2] Aguilar-Pérez C. Gracia B. Rodrigues L. Vitoria A. Cebrián R. Deboosère N. (2018). Synergy between circular bacteriocin AS-48 and ethambutol against *Mycobacterium tuberculosis*. *Antimicrob. Agents Chemother.* 62:e00359-18. 10.1128/AAC.00359-18 29987141 PMC6125546

[B3] Ahlberg S. H. Joutsjoki V. Korhonen H. J. (2015). Potential of lactic acid bacteria in aflatoxin risk mitigation. *Int. J. Food Microbiol.* 207 87–102. 10.1016/j.ijfoodmicro.2015.04.042 26001523

[B4] Al Atya A. K. Belguesmia Y. Chataigne G. Ravallec R. Vachée A. Szunerits S. (2016). Anti-MRSA activities of enterocins DD28 and DD93 and evidences on their role in the inhibition of biofilm formation. *Front. Microbiol.* 7:817. 10.3389/fmicb.2016.00817 27303396 PMC4886693

[B5] Al kassaa I. Hober D. Chihib N. Drider D. (2014). Antiviral potential of lactic acid bacteria and their bacteriocins. *Probiotics Antimicrob. Proteins* 6 177–185. 10.1007/s12602-014-9162-6 24880436

[B6] Allen A. Flemström G. (2005). Gastroduodenal mucus bicarbonate barrier: protection against acid and pepsin. *Am. J. Physiol. Cell Physiol.* 288 C1–C19. 10.1152/ajpcell.00102.2004 15591243

[B7] Allen H. K. Levine U. Y. Looft T. Bandrick M. Casey T. A. (2013). Treatment, promotion, commotion: antibiotic alternatives in food-producing animals. *Trends Microbiol.* 21 114–119. 10.1016/j.tim.2012.11.001 23473629

[B8] Alloui M. N. Szczurek W. Światkiewicz S. (2013). The usefulness of prebiotics and probiotics in modern poultry nutrition: a review. *Ann. Anim. Sci.* 13 17–32. 10.2478/v10220-012-0055-x

[B9] Al-Saiady M. Y. (2010). Effect of probiotic bacteria on immunoglobulin G concentration and other blood componenets of newborn calves. *J. Anim. Vet. Sci.* 9 604–609. 10.3923/javaa.2010.604.609

[B10] American Meat Institution (2013). *The Facts About Antibiotics in Livestock & Poultry Production.* Available at: https://www.meatinstitute.org/index.php?ht=a/GetDocumentAction/i/99943

[B11] Anacarso I. Messi P. Condó C. Iseppi R. Bondi M. Sabia C. (2014). A bacteriocin-like substance produced from *Lactobacillus pentosus* 39 is a natural antagonist for the control of *Aeromonas hydrophila* and *Listeria monocytogenes* in fresh salmon fillets. *LWT – Food Sci. Technol.* 55 604–611. 10.1016/j.lwt.2013.10.012

[B12] Andersen A. D. Cilieborg M. S. Lauridsen C. Morkbak A. L. Sangild P. T. (2017). Supplementation with *Lactobacillus paracasei* or *Pediococcus pentosaceus* does not prevent diarrhoea in neonatal pigs infected with *Escherichia coli* F18. *Br. J. Nutr.* 118 109–120. 10.1017/S000711451700160X 28720151

[B13] Anderson R. C. Cookson A. L. Mcnabb W. C. Park Z. Mccann M. J. Kelly W. J. (2010). *Lactobacillus plantarum* MB452 enhances the function of the intestinal barrier by increasing the expression levels of genes involved in tight junction formation. *BMC Microbiol.* 10:316. 10.1186/1471-2180-10-316 21143932 PMC3004893

[B14] Angelakis E. (2017). Weight gain by gut microbiota manipulation in productive animals. *Microb. Pathog.* 106 162–170. 10.1016/j.micpath.2016.11.002 27836763

[B15] Arakawa K. Yoshida S. Aikawa H. Hano C. Bolormaa T. Burenjargal S. (2016). Production of a bacteriocin-like inhibitory substance by *Leuconostoc mesenteroides* subsp. *dextranicum* 213M0 isolated from Mongolian fermented mare milk, airag. *Anim. Sci. J.* 87 449–456. 10.1111/asj.12445 26388181

[B16] Araújo C. Muñoz-Atienza E. Poeta P. Igrejas G. Hernández P. E. Herranz C. (2016). Characterization of *Pediococcus acidilactici* strains isolated from rainbow trout (*Oncorhynchus mykiss*) feed and larvae: safety, DNA fingerprinting, and bacteriocinogenicity. *Dis. Aquat. Organ.* 119 129–143. 10.3354/dao02992 27137071

[B17] Arena M. P. Capozzi V. Russo P. Drider D. Spano G. Fiocco D. (2018a). Immunobiosis and probiosis: antimicrobial activity of lactic acid bacteria with a focus on their antiviral and antifungal properties. *Appl. Microbiol. Biotechnol.* 102 9949–9958. 10.1007/s00253-018-9403-9 30280241

[B18] Arena M. P. Elmastour F. Sane F. Drider D. Fiocco D. Spano G. (2018b). Inhibition of coxsackievirus B4 by *Lactobacillus plantarum*. *Microbiol. Res.* 210 59–64. 10.1016/j.micres.2018.03.008 29625659

[B19] Arruda P. H. Madson D. M. Ramirez A. Rowe E. W. Songer J. G. (2016). Bacterial probiotics as an aid in the control of *Clostridium difficile* disease in neonatal pigs. *Can. Vet. J.* 57 183–188. 26834271 PMC4712999

[B20] Asgari F. Madjd Z. Falak R. Bahar M. A. Nasrabadi M. H. Raiani M. (2016). Probiotic feeding affects T cell populations in blood and lymphoid organs in chickens. *Benef. Microbes* 7 669–675. 10.3920/BM2016.0014 27349931

[B21] Ashraf R. Shah N. P. (2014). Immune system stimulation by probiotic microorganisms. *Crit. Rev. Food Sci. Nutr.* 54 938–956. 10.1080/10408398.2011.619671 24499072

[B22] Baldwin S. Hughes R. J. Hao Van T. T. Moore R. J. Stanley D. (2018). At-hatch administration of probiotic to chickens can introduce beneficial changes in gut microbiota. *PLoS One* 13:e0194825. 10.1371/journal.pone.0194825 29570728 PMC5865720

[B23] Banerjee G. Ray A. K. (2017). The advancement of probiotics research and its application in fish farming industries. *Res. Vet. Sci.* 115 66–77. 10.1016/j.rvsc.2017.01.016 28157611

[B24] Beck B. R. Kim D. Jeon J. Lee S. M. Kim H. K. Kim O. J. (2015). The effects of combined dietary probiotics *Lactococcus lactis* BFE920 and *Lactobacillus plantarum* FGL0001 on innate immunity and disease resistance in olive flounder (*Paralichthys olivaceus*). *Fish Shellfish Immunol.* 42 177–183. 10.1016/j.fsi.2014.10.035 25449382

[B25] Belguesmia Y. Choiset Y. Prévost H. Dalgalarrondo M. Chobert J. Drider D. (2010). Partial purification and characterization of the mode of action of enterocin S37: a bacteriocin produced by *Enterococcus faecalis* S37 isolated from poultry feces. *J. Environ. Public Health* 2010:986460. 10.1155/2010/986460 20811593 PMC2929494

[B26] Ben Lagha A. Haas B. Gottschalk M. Grenier D. (2017). Antimicrobial potential of bacteriocins in poultry and swine production. *Vet. Res.* 48 1–12. 10.1186/s13567-017-0425-6 28399941 PMC5387282

[B27] Boyd J. West J. W. Bernard J. K. Aoac G. Bergner H. Kijora C. (2011). Effects of the addition of direct-fed microbials and glycerol to the diet of lactating dairy cows on milk yield and apparent efficiency of yield. *J. Dairy Sci.* 94 4616–4622. 10.3168/jds.2010-3984 21854934

[B28] Brisbin J. T. Gong J. Orouji S. Esufali J. Mallick A. I. Parvizi P. (2011). Oral treatment of chickens with Lactobacilli influences elicitation of immune responses. *Clin. Vaccine Immunol.* 18 1447–1455. 10.1128/CVI.05100-11 21734067 PMC3165221

[B29] Brown K. Uwiera R. R. E. Kalmokoff M. L. Brooks S. P. J. Inglis G. D. (2017). Antimicrobial growth promoter use in livestock: a requirement to understand their modes of action to develop effective alternatives. *Int. J. Antimicrob. Agents* 49 12–24. 10.1016/j.ijantimicag.2016.08.006 27717740

[B30] Butaye P. Devriese L. A. Haesebrouck F. (2003). Antimicrobial growth promoters used in animal feed: effects of less well known antibiotics on gram-positive bacteria. *Clin. Microbiol. Rev.* 16 175–188. 10.1128/CMR.16.2.175-188.2003 12692092 PMC153145

[B31] Callaway T. R. Edrington T. S. Anderson R. C. Harvey R. B. Genovese K. J. Kennedy C. N. (2008). Probiotics, prebiotics and competitive exclusion for prophylaxis against bacterial disease. *Anim. Health Res. Rev.* 9 217–225. 10.1017/S1466252308001540 19102792

[B32] Caly D. L. Chevalier M. Flahaut C. Cudennec B. Al Atya A. K. Chataigné G. (2017). The safe enterocin DD14 is a leaderless two-peptide bacteriocin with anti-*Clostridium perfringens* activity. *Int. J. Antimicrob. Agents* 49 282–289. 10.1016/j.ijantimicag.2016.11.016 28104423

[B33] Caly D. L. D’Inca R. Auclair E. Drider D. (2015). Alternatives to antibiotics to prevent necrotic enteritis in broiler chickens: a microbiologist’s perspective. *Front. Microbiol.* 6:1336 10.3389/fmicb.2015.01336 PMC4664614 26648920

[B34] Cao L. T. Wu J. Q. Xie F. Hu S. H. Mo Y. (2007). Efficacy of nisin in treatment of clinical mastitis in lactating dairy cows. *J. Dairy Sci.* 90 3980–3985. 10.3168/jds.2007-0153 17639009

[B35] Carnevali O. Maradonna F. Gioacchini G. (2017). Integrated control of fish metabolism, wellbeing and reproduction: the role of probiotic. *Aquaculture* 472 144–155. 10.1016/j.aquaculture.2016.03.037

[B36] Carter A. Adams M. La Ragione R. M. Woodward M. J. (2017). Colonisation of poultry by *Salmonella* Enteritidis S1400 is reduced by combined administration of *Lactobacillus salivarius* 59 and *Enterococcus faecium* PXN-33. *Vet. Microbiol.* 199 100–107. 10.1016/j.vetmic.2016.12.029 28110775

[B37] Cavera V. L. Arthur T. D. Kashtanov D. Chikindas M. L. (2015). Bacteriocins and their position in the next wave of conventional antibiotics. *Int. J. Antimicrob. Agents* 46 494–501. 10.1016/j.ijantimicag.2015.07.011 26341839

[B38] Chaucheyras-Durand F. Durand H. (2010). Probiotics in animal nutrition and health. *Benef. Microbes* 1 3–9. 10.3920/BM2008.1002 21840795

[B39] Cheng G. Hao H. Xie S. Wang X. Dai M. Huang L. (2014). Antibiotic alternatives: the substitution of antibiotics in animal husbandry? *Front. Microbiol.* 5:217. 10.3389/fmicb.2014.00217 24860564 PMC4026712

[B40] Chi H. Holo H. (2018). Synergistic antimicrobial activity between the broad spectrum bacteriocin garvicin KS and nisin, farnesol and polymyxin B against Gram-positive and Gram-negative bacteria. *Curr. Microbiol.* 75 272–277. 10.1007/s00284-017-1375-y 29058043 PMC5809525

[B41] Chikindas M. L. Weeks R. Drider D. Chistyakov V. A. Dicks L. M. T. (2018). Functions and emerging applications of bacteriocins. *Curr. Opin. Biotechnol.* 49 23–28. 10.1016/j.copbio.2017.07.011 28787641 PMC5799035

[B42] Czaplewski L. Bax R. Clokie M. Dawson M. Fairhead H. Fischetti V. A. (2016). Alternatives to antibiotics-a pipeline portfolio review. *Lancet Infect. Dis.* 16 239–251. 10.1016/S1473-3099(15)00466-1 26795692

[B43] Damayanti E. Istiqomah L. Saragih J. E. Purwoko T. and Sardjono. (2017). Characterization of lactic acid bacteria as poultry probiotic candidates with aflatoxin B1 binding activities. *IOP Conf. Ser. Earth Environ. Sci.* 101:012030 10.1088/1755-1315/

[B44] Diepers A. Krömker V. Zinke C. Wente N. Pan L. Paulsen K. (2017). In vitro ability of lactic acid bacteria to inhibit mastitis-causing pathogens. *Sustain. Chem. Pharm.* 5 84–92. 10.1016/j.scp.2016.06.002 22077995

[B45] Dittoe D. K. Ricke S. C. Kiess A. S. (2018). Organic acids and potential for modifying the avian gastrointestinal tract and reducing pathogens and disease. *Front. Vet. Sci.* 5:216. 10.3389/fvets.2018.00216 30238011 PMC6136276

[B46] Dowarah R. Verma A. K. Agarwal N. (2017). The use of *Lactobacillus* as an alternative of antibiotic growth promoters in pigs: a review. *Anim. Nutr.* 3 1–6. 10.1016/j.aninu.2016.11.002 29767055 PMC5941084

[B47] Dowarah R. Verma A. K. Agarwal N. Singh P. (2018). Efficacy of species-specific probiotic *Pediococcus acidilactici* FT28 on blood biochemical profile, carcass traits and physicochemical properties of meat in fattening pigs. *Res. Vet. Sci.* 117 60–64. 10.1016/j.rvsc.2017.11.011 29179030

[B48] Drider D. Bendali F. Naghmouchi K. Chikindas M. L. (2016). Bacteriocins: not only antibacterial agents. *Probiotics Antimicrob. Proteins* 8 177–182. 10.1007/s12602-016-9223-0 27481236

[B49] Drider D. Rebuffat S. (2011). *Prokaryotic Antimicrobial Peptides: From Genes to Applications.* New York, NY: Springer 10.1007/978-1-4419-7692-5

[B50] Drissi F. Raoult D. Merhej V. (2017). Metabolic role of lactobacilli in weight modification in humans and animals. *Microb. Pathog.* 106 182–194. 10.1016/j.micpath.2016.03.006 27033001

[B51] El-Nezami H. Kankaanpaa P. Salminen S. Ahokas J. (1998). Ability of dairy strains of lactic acid bacteria to bind a common food carcinogen, aflatoxin B1. *Food Chem. Toxicol.* 36 321–326. 10.1016/S0278-6915(97)00160-9 9651049

[B52] El-NezamiH.PolychronakiN.SalminenS.MykkaH. (2002). Binding rather than metabolism may explain the interaction of two food-grade *Lactobacillus* strains with Zearalenone and its derivative 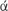 ì-Zearaleno. *Appl. Environ. Microbiol.* 68 3545–3549. 10.1128/AEM.68.7.354512089040PMC126820

[B53] Elshaghabee F. M. F. Bockelmann W. Meske D. Vrese M. D. Walte H. G. Schrezenmeir J. (2016). Ethanol production by selected intestinal microorganisms and lactic acid bacteria growing under different nutritional conditions. *Front. Microbiol.* 7:47. 10.3389/fmicb.2016.00047 26858714 PMC4732544

[B54] ESVAC (2017). *Sales of Veterinary Antimicrobial Agents in 30 European Countries in 2015. Trends from 2010 to 2015 (EMA/184855/2017).*

[B55] Ewaschuk J. B. Diaz H. Meddings L. Diederichs B. Dmytrash A. Backer J. (2008). Secreted bioactive factors from *Bifidobacterium infantis* enhance epithelial cell barrier function. *Am. J. Physiol. Liver Physiol.* 295 1025–1034. 10.1152/ajpgi.90227.2008 18787064

[B56] FAO (2013). *Poultry Development Review.* Rome: FAO.

[B57] FAO (2016). “Probiotics in animal nutrition – Production, impact and regulation,” in *Makkar FAO Animal Production and Health Paper No. 179*, ed. Harinder P. S. (Rome: FAO). 10.3920/BM2008.1002

[B230] FAOSTAT (2013). *Food and Agriculture Organization of the United Nations, Statistics Division*. Available at: http://www.fao.org/faostat/en/#data/QL (accessed July 22, 2018).

[B58] FAO/WHO (2002). *Guidelines for the Evaluation of Probiotics in Food.* Rome: FAO 10.1111/j.1469-0691.2012.03873

[B59] Forte C. Moscati L. Acuti G. Mugnai C. Franciosini M. P. Costarelli S. (2016). Effects of dietary *Lactobacillus acidophilus* and *Bacillus subtilis* on laying performance, egg quality, blood biochemistry and immune response of organic laying hens. *J. Anim. Physiol. Anim. Nutr.* 100 977–987. 10.1111/jpn.12408 26614687

[B60] Frese S. A. Benson A. K. Tannock G. W. Loach D. M. Kim J. Zhang M. (2011). The evolution of host specialization in the vertebrate gut symbiont *Lactobacillus reuteri*. *PLoS Genet.* 7:e1001314. 10.1371/journal.pgen.1001314 21379339 PMC3040671

[B61] Frizzo L. S. Soto L. P. Zbrun M. V. Bertozzi E. Sequeira G. Armesto R. R. (2010). Lactic acid bacteria to improve growth performance in young calves fed milk replacer and spray-dried whey powder. *Anim. Feed Sci. Technol.* 157 159–167. 10.1016/j.anifeedsci.2010.03.005

[B62] Fry J. P. Mailloux N. A. Love D. C. Milli M. C. Cao L. (2018). Feed conversion efficiency in aquaculture: do we measure it correctly? *Environ. Res. Lett.* 13:024017 10.1088/1748-9326/aaa273

[B63] Gaggìa F. Mattarelli P. Biavati B. (2010). Probiotics and prebiotics in animal feeding for safe food production. *Int. J. Food Microbiol.* 141 S15–S28. 10.1016/j.ijfoodmicro.2010.02.031 20382438

[B64] Gao X. Zhang M. Li X. Yin H. Fucun W. Ying L. (2018). The effects of feeding *Lactobacillus pentosus* on growth, immunity, and disease resistance in *Haliotis discus* hannai Ino. *Fish Shellfish Immunol.* 482 221–230. 10.1016/j.fsi.2018.04.010 29626669

[B65] García-Quintáns N. Repizo G. Martín M. Magni C. López P. (2008). Activation of the diacetyl/acetoin pathway in *Lactococcus lactis* subsp. *lactis* bv. *diacetylactis* CRL264 by acidic growth. *Appl. Environ. Microbiol.* 74 1988–1996. 10.1128/AEM.01851-07 18245243 PMC2292580

[B66] Gardiner G. E. Rea M. C. Riordan B. O. Connor P. O. Morgan S. M. Lawlor P. G. (2007). Fate of the two-component lantibiotic lacticin 3147 in the gastrointestinal tract. *Appl. Environ. Microbiol.* 73 7103–7109. 10.1128/AEM.01117-07 17766459 PMC2074984

[B67] Giannenas I. Papadopoulos E. Tsalie E. Triantafillou E. Henikl S. Teichmann K. (2012). Assessment of dietary supplementation with probiotics on performance, intestinal morphology and microflora of chickens infected with *Eimeria tenella*. *Vet. Parasitol.* 188 31–40. 10.1016/j.vetpar.2012.02.017 22459110

[B68] Gibson G. R. Probert H. M. Van Loo J. Rasrall R. A. Roberfroid M. B. (2004). Dietary modulation of the human colonic microbiota: introducing the concept of prebiotics. *Nutr. Res. Rev.* 17 259–275. 10.1079/NRR200479 19079930

[B69] Gioacchini G. Giorgini E. Merrifield D. L. Hardiman G. Borini A. Vaccari L. (2012). Probiotics can induce follicle maturational competence: the *Danio rerio* case. *Biol. Reprod.* 86 1–11. 10.1095/biolreprod.111.094243 22088919 PMC3316265

[B70] Gioacchini G. Maradonna F. Lombardo F. Bizzaro D. Olivotto I. Carnevali O. (2010). Increase of fecundity by probiotic administration in zebrafish (*Danio rerio*). *Reprod. Res.* 140 953–959. 10.1530/REP-10-0145 20833753

[B71] Gómez N. C. Ramiro J. M. Quecan B. X. de Melo Franco B. D. (2016). Use of potential probiotic lactic acid bacteria (LAB) biofilms for the control of *Listeria monocytogenes*, *Salmonella* Typhimurium, and *Escherichia coli* O157:H7 biofilms formation. *Front. Microbiol.* 7:863. 10.3389/fmicb.2016.00863 27375584 PMC4901071

[B72] Gough R. Rubio R. C. Connor P. M. O. Crispie F. Brodkorb A. Miao S. (2018). Oral delivery of nisin in resistant starch based matrices alters the gut microbiota in mice. *Front. Microbiol.* 9:1186. 10.3389/fmicb.2018.01186 29963017 PMC6013561

[B73] Grande Burgos M. Pulido R. Del Carmen López Aguayo M. Gálvez A. Lucas R. (2014). The cyclic antibacterial peptide enterocin AS-48: isolation, mode of action, and possible food applications. *Int. J. Mol. Sci.* 15 22706–22727. 10.3390/ijms151222706 25493478 PMC4284732

[B74] Gratz S. Wu Q. K. El-Nezami H. Juvonen R. O. Mykkänen H. Turner P. C. (2007). *Lactobacillus rhamnosus* strain GG reduces aflatoxin B1transport, metabolism, and toxicity in Caco-2 cells. *Appl. Environ. Microbiol.* 73 3958–3964. 10.1128/AEM.02944-06 17449679 PMC1932713

[B75] Grilli E. Messina M. R. Catelli E. Morlacchini M. Piva A. (2009). Pediocin A improves growth performance of broilers challenged with *Clostridium perfringens*. *Poult. Sci.* 88 2152–2158. 10.3382/ps.2009-00160 19762869

[B76] Guerra-Ordaz A. A. González-Ortiz G. La Ragione R. M. Woodward M. J. Collins J. W. Pérez J. F. (2014). Lactulose and *Lactobacillus plantarum*, a potential complementary synbiotic to control postweaning colibacillosis in piglets. *Appl. Environ. Microbiol.* 80 4879–4886. 10.1128/AEM.00770-14 24907322 PMC4135760

[B77] Guo S. Liu D. Zhang B. Li Z. Li Y. Ding B. (2017). Two Lactobacillus species inhibit the growth and α-toxin production of *Clostridium perfringens* and induced proinflammatory factors in chicken intestinal epithelial cells in vitro. *Front. Microbiol.* 8:2081 10.3389/fmicb.2017.02081 PMC5661052 29118744

[B78] Gupta P. Porwal K. S. S. M. Joshi M. (2015). Biological performance of female calves fed diets supplemented with different strains of lactobacilli. *Int. J. Sci. Environ. Technol.* 4 1181–1187.

[B79] Hai N. V. (2015). The use of probiotics in aquaculture. *J. Appl. Microbiol.* 119 917–935. 10.1111/jam.12886 26119489

[B80] Han J. Wang Y. Song D. Lu Z. Dong Z. Miao H. (2018). Effects of *Clostridium butyricum* and *Lactobacillus plantarum* on growth performance, immune function and volatile fatty acid level of caecal digesta in broilers. *Food Agric. Immunol.* 29 797–807. 10.1080/09540105.2018.1457013

[B81] Hardy H. Harris J. Lyon E. Beal J. Foey A. D. (2013). Probiotics, prebiotics and immunomodulation of gut mucosal defences: homeostasis and immunopathology. *Nutrients* 5 1869–1912. 10.3390/nu5061869 23760057 PMC3725482

[B82] Haskard C. A. El-nezami H. S. Kankaanpää P. E. Salminen S. Ahokas J. T. (2001). Surface binding of aflatoxin B1 by lactic acid bacteria. *Appl. Environ. Microbiol.* 67 3086–3091. 10.1128/AEM.67.7.3086 11425726 PMC92985

[B83] Helal M. M. Hashem A. M. Ghobashy M. O. I. Shalaby A. S. G. (2016). Some physiological and biological studies on reuterin production from *Lactobacillus reuteri*. *J. Probiotics Health* 4:1000156 10.4172/2329-8901.1000156

[B84] Hume M. E. Corrier D. E. Ivie G. W. Deloach J. R. (1993). Metabolism of [14C]propionic acid in broiler chicks. *Poult. Sci.* 72 786–793. 10.3382/ps.0720786 8502603

[B85] ILCA (1990). *Livestock Systems Research Manual: Working Paper 1.* London: ILCA.

[B86] Ingram L. O. (1989). Ethanol tolerance in bacteria. *Crit. Rev. Biotechnol.* 9 305–319. 10.3109/07388558909036741 2178781

[B87] Iwasaki A. Medzhitov R. (2015). Control of adaptive immunity by the innate immune system. *Nat. Immunol.* 16:343. 10.1038/ni.3123 25789684 PMC4507498

[B88] Jiang H. Li P. Gu Q. (2016). Heterologous expression and purification of plantaricin NC8, a two-peptide bacteriocin against *Salmonella* spp. from *Lactobacillus plantarum* ZJ316. *Protein Expr. Purif.* 127 28–34. 10.1016/j.pep.2016.06.013 27373940

[B89] Juncioni de Arauz L. Faustino Jozala A. Gava Mazzola P. Vessoni Penna T. C. (2009). Nisin biotechnological production and application: a review. *Trends Food Sci. Technol.* 20 146–154. 10.1016/j.tifs.2009.01.056

[B90] Józefiak D. Sip A. Rutkowski A. Rawski M. Kaczmarek S. Wołuń-Cholewa M. (2012). Lyophilized *Carnobacterium divergens* AS7 bacteriocin preparation improves performance of broiler chickens challenged with *Clostridium perfringens*. *Poult. Sci.* 91 1899–1907. 10.3382/ps.2012-02151 22802184

[B91] Kabir S. M. L. (2009). The role of probiotics in the poultry industry. *Int. J. Mol. Sci.* 10 3531–3546. 10.3390/ijms10083531 20111681 PMC2812824

[B92] Karpiński T. M. Szkaradkiewicz A. K. (2013). Characteristic of bacteriocines and their application. *Polish J. Microbiol.* 62 223–235. 24459827

[B93] Kers J. G. Velkers F. C. Fischer E. A. J. Hermes G. D. A. Stegeman J. A. Smidt H. (2018). Host and environmental factors affecting the intestinal microbiota in chickens. *Front. Microbiol.* 9:235. 10.3389/fmicb.2018.00235 29503637 PMC5820305

[B94] Kiczorowska B. Samolińska W. Al-Yasiry A. R. M. Kiczorowski P. Winiarska-Mieczan A. (2017a). The natural feed additives as immunostimulants in monogastric animal nutrition – a review. *Ann. Anim. Sci.* 17 605–625. 10.1515/aoas-2016-0076

[B95] Kiczorowska B. Samolińska W. Marczuk J. Winiarska-Mieczan A. Klebaniuk R. Kowalczuk-Vasilev E. (2017b). Comparative effects of organic, traditional, and intensive production with probiotics on the fatty acid profile of cow’s milk. *J. Food Compos. Anal.* 63 157–163. 10.1016/j.jfca.2017.08.002

[B96] Kogut M. H. (2014). Perspectives and research challenges in veterinary infectious diseases. *Front. Vet. Sci.* 1:21 10.3389/fvets.2014.00021 PMC4668856 26664920

[B97] Krajmalnik-Brown R. Ilhan Z. E. Kang D. W. DiBaise J. K. (2012). Effects of gut microbes on nutrient absorption and energy regulation. *Nutr. Clin. Pract.* 27 201–214. 10.1177/0884533611436116.Effects 22367888 PMC3601187

[B98] La Ragione R. M. Narbad A. Gasson M. J. Woodward M. J. (2004). In vivo characterization of *Lactobacillus johnsonii* FI9785 for use as a defined competitive exclusion agent against bacterial pathogens in poultry. *Lett. Appl. Microbiol.* 38 197–205. 10.1111/j.1472-765X.2004.01474.x 14962040

[B99] Lan R. X. Koo J. M. Kim I. H. (2016). Effects of *Lactobacillus acidophilus* supplementation in different energy and nutrient density diets on growth performance, nutrient digestibility, blood characteristics, fecal microbiota shedding, and fecal noxious gas emission in weaning pigs. *Anim. Feed Sci. Technol.* 219 181–188. 10.1016/j.anifeedsci.2016.06.018

[B100] Laxminarayan R. Van Boeckel T. Teillant A. (2015). *The Economic Costs of Withdrawing Antimicrobial Growth Promoters From the Livestock Sector.* OECD Food, Agriculture and Fisheries Papers, No. 78. Paris: OECD Publishing 10.1787/5js64kst5wvl-en

[B101] Layton S. L. Hernandez-velasco X. Chaitanya S. Xavier J. Menconi A. Latorre J. D. (2013). The effect of a Lactobacillus-based probiotic for the control of necrotic enteritis in broilers. *Food Nutr. Sci.* 4 1–7. 10.4236/fns.2013.411A001

[B102] Lebeer S. Vanderleyden J. De Keersmaecker S. C. (2010). Host interactions of probiotic bacterial surface molecules: comparison with commensals and pathogens. *Nat. Rev. Microbiol.* 8 171–184. 10.1038/nrmicro2297 20157338

[B103] Lebeer S. Vanderleyden J. De Keersmaecker S. C. J. (2008). Genes and molecules of lactobacilli supporting probiotic action. *Microbiol. Mol. Biol. Rev.* 72 728–764. 10.1128/MMBR.00017-08 19052326 PMC2593565

[B104] Lebel G. Piché F. Frenette M. Gottschalk M. Grenier D. (2013). Antimicrobial activity of nisin against the swine pathogen *Streptococcus suis* and its synergistic interaction with antibiotics. *Peptides* 50 19–23. 10.1016/j.peptides.2013.09.014 24096107

[B105] Lee O. N. Lyu S. R. Wang R. C. Weng C. F. Chen B. J. (2011). Exhibit differential functions of various antibiotic growth promoters in broiler growth, immune response and gastrointestinal physiology. *Int. J. Poult. Sci.* 10 216–220. 10.3923/ijps.2011.216.220

[B106] Leyva-Madrigal K. Y. Luna-González A. Escobedo-Bonilla C. M. Fierro-Coronado J. A. Maldonado-Mendoza I. E. (2011). Screening for potential probiotic bacteria to reduce prevalence of WSSV and IHHNV in whiteleg shrimp (*Litopenaeus vannamei*) under experimental conditions. *Aquaculture* 32 16–22. 10.1016/j.aquaculture.2011.09.033

[B107] Li H. Duan C. Zhao Y. Gao L. Niu C. Xu J. (2017). Reduction of aflatoxin B1 toxicity by *Lactobacillus plantarum* C88: a potential probiotic strain isolated from Chinese traditional fermented food “Tofu”. *PLoS One* 12:e0170109. 10.1371/journal.pone.0170109 28129335 PMC5271326

[B108] Li H. Zhang L. Chen L. Zhu Q. Wang W. Qiao J. (2016). *Lactobacillus acidophilus* alleviates the inflammatory response to enterotoxigenic *Escherichia coli* K88 via inhibition of the NF- κ B and p38 mitogen-activated protein kinase signaling pathways in piglets. *BMC Microbiol.* 16:273. 10.1186/s12866-016-0862-9 27832756 PMC5105324

[B109] Liao S. F. Nyachoti C. M. (2017). Using probiotics to improve swine gut health and nutrient utilization. *Anim. Nutr.* 3 331–343. 10.1016/j.aninu.2017.06.007 29767089 PMC5941265

[B110] Liew W. P. P. Nurul-Adilah Z. Than L. T. L. Mohd-Redzwan S. (2018). The binding efficiency and interaction of *Lactobacillus casei* Shirota toward aflatoxin B1. *Front. Microbiol.* 9:1503. 10.3389/fmicb.2018.01503 30042748 PMC6048233

[B111] Lindgren S. E. Dobrogosz W. J. (1990). Antagonistic activities of lactic acid bacteria in food and feed fermentations. *FEMS Microbiol. Lett.* 87 149–164. 10.1016/0378-1097(90)90703-S 2125429

[B112] Liu H. Ji H. F. Zhang D. Y. Wang S. X. Wang J. Shan D. C. (2015). Effects of *Lactobacillus brevis* preparation on growth performance, fecal microflora and serum profile in weaned pigs. *Livest. Sci.* 178 251–254. 10.1016/j.livsci.2015.06.002

[B113] Liu L. Zhang W. Song Y. Wang W. Zhang Y. Wang T. (2017). Recombinant *Lactococcus lactis* co-expressing OmpH of an M cell-targeting ligand and IBDV-VP2 protein provide immunological protection in chickens. *Vaccine* 36 729–735. 10.1016/j.vaccine.2017.12.027 29289381

[B114] Luongo D. Miyamoto J. Bergamo P. Nazzaro F. Baruzzi F. Sashihara T. (2013). Differential modulation of innate immunity in vitro by probiotic strains of *Lactobacillus gasseri*. *BMC Microbiol.* 13:298. 10.1186/1471-2180-13-298 24365457 PMC3879436

[B115] Madsen K. L. (2012). Enhancement of epithelial barrier function by probiotics. *J. Epithel. Biol. Pharmacol.* 5 55–59. 10.2174/1875044301205010055

[B116] Marshall B. M. Levy S. B. (2011). Food animals and antimicrobials: impacts on human health. *Clin. Microbiol. Rev.* 24 718–733. 10.1128/CMR.00002-11 21976606 PMC3194830

[B117] Martínez Cruz P. Ibáñez A. L. Monroy Hermosillo O. A. Ramírez Saad H. C. (2012). Use of probiotics in aquaculture. *ISRN Microbiol.* 2012 1–13. 10.5402/2012/916845 23762761 PMC3671701

[B118] Mathias A. Duc M. Favre L. Benyacoub J. Blum S. Corthesy B. (2010). Potentiation of polarized intestinal Caco-2 cell responsiveness to probiotics complexed with secretory IgA. *J. Biol. Chem.* 285 33906–33913. 10.1074/jbc.M110.135111 20729211 PMC2962490

[B119] Messaoudi S. Madi A. Prévost H. Feuilloley M. Manai M. Dousset X. (2012). *In vitro* evaluation of the probiotic potential of *Lactobacillus salivarius* SMXD51. *Anaerobe* 18 584–589. 10.1016/j.anaerobe.2012.10.004 23122647

[B120] Mikulski D. Jankowski J. Naczmanski J. Mikulska M. Demey V. (2012). Effects of dietary probiotic (*Pediococcus acidilactici*) supplementation on performance, nutrient digestibility, egg traits, egg yolk cholesterol, and fatty acid profile in laying hens. *Poult. Sci.* 91 2691–2700. 10.3382/ps.2012-02370 22991559

[B121] Million M. Angelakis E. Paul M. Armougom F. Leibovici L. Raoult D. (2012). Comparative meta-analysis of the effect of *Lactobacillus* species on weight gain in humans and animals. *Microb. Pathog.* 53 100–108. 10.1016/j.micpath.2012.05.007 22634320

[B122] Mitchell C. Fredricks D. Agnew K. Hitti J. (2015). Hydrogen peroxide-producing lactobacilli are associated with lower levels of vaginal interleukin-1β, independent of bacterial vaginosis. *Sex. Transm. Dis.* 42 358–363. 10.1097/OLQ.0000000000000298 26222747 PMC4520248

[B123] Mookiah S. Sieo C. C. Ramasamy K. Abdullah N. Ho Y. W. (2014). Effects of dietary prebiotics, probiotic and synbiotics on performance, caecal bacterial populations and caecal fermentation concentrations of broiler chickens. *J. Sci. Food Agric.* 94 341–348. 10.1002/jsfa.6365 24037967

[B124] M’Sadeq S. A. Wu S. Swick R. A. Choct M. (2015). Towards the control of necrotic enteritis in broiler chickens with in-feed antibiotics phasing-out worldwide. *Anim. Nutr.* 1 1–11. 10.1016/j.aninu.2015.02.004 29766984 PMC5884463

[B125] Murphy D. Ricci A. Auce Z. Beechinor J. G. Bergendahl H. Breathnach R. (2017). EMA and EFSA Joint Scientific Opinion on measures to reduce the need to use antimicrobial agents in animal husbandry in the European Union, and the resulting impacts on food safety (RONAFA). *EFSA J.* 15:e04666 10.2903/j.efsa.2017.4666 PMC7010070 32625259

[B126] Naghmouchi K. Baah J. Hober D. Jouy E. Rubrecht C. Sané F. (2013). Synergistic effect between colistin and bacteriocins in controlling Gram-negative pathogens and their potential to reduce antibiotic toxicity in mammalian epithelial cells. *Antimicrob. Agents Chemother.* 57 2719–2725. 10.1128/AAC.02328-12 23571533 PMC3716138

[B127] Naghmouchi K. Belguesmia Y. Baah J. Teather R. Drider D. (2011). Antibacterial activity of class I and IIa bacteriocins combined with polymyxin E against resistant variants of *Listeria monocytogenes* and *Escherichia coli*. *Res. Microbiol.* 162 99–107. 10.1016/j.resmic.2010.09.014 20868743

[B128] Naghmouchi K. Drider D. Baah J. Teather R. (2010). Nisin A and polymyxin B as synergistic inhibitors of Gram-positive and Gram-negative bacteria. *Probiotics Antimicrob. Proteins* 2 98–103. 10.1007/s12602-009-9033-8 26781118

[B129] Naqid I. A. Owen J. P. Maddison B. C. Gardner D. S. Foster N. Tchórzewska M. A. (2015). Prebiotic and probiotic agents enhance antibody-based immune responses to *Salmonella* Typhimurium infection in pigs. *Anim. Feed Sci. Technol.* 201 57–65. 10.1016/j.anifeedsci.2014.12.005

[B130] Newell D. G. Koopmans M. Verhoef L. Duizer E. Aidara-Kane A. Sprong H. (2010). Food-borne diseases - The challenges of 20 years ago still persist while new ones continue to emerge. *Int. J. Food Microbiol.* 139 S3–S15. 10.1016/j.ijfoodmicro.2010.01.021 20153070 PMC7132498

[B131] Nguyen T. L. Park C.-I. Kim D.-H. (2017). Improved growth rate and disease resistance in olive flounder, *Paralichthys olivaceus*, by probiotic *Lactococcus lactis* WFLU12 isolated from wild marine fish. *Aquaculture* 471 113–120. 10.1016/j.aquaculture.2017.01.008

[B132] Niewold T. A. (2007). The nonantibiotic anti-inflammatory effect of antimicrobial growth promoters, the real mode of action? A hypothesis. *Poult. Sci.* 86 605–609. 10.1093/ps/86.4.605 17369528

[B133] Nybom S. M. K. Salminen S. J. Meriluoto J. A. O. (2007). Removal of microcystin-LR by strains of metabolically active probiotic bacteria. *FEMS Microbiol. Lett.* 270 27–33. 10.1111/j.1574-6968.2007.00644.x 17263839

[B134] O’Bryan C. A. Crandall P. G. Ricke S. C. Ndahetuye J. B. (2014). *Lactic Acid Bacteria (LAB) as Antimicrobials in Food Products: Types and Mechanisms of Action.* New York City, NY: Elsevier Ltd. 10.1016/B978-1-78242-034-7.00006-2

[B135] OECD/FAO (2018). *OECD - FAO Agricultural Outlook 2018 – 2027.* Paris: OECD Publishing 10.1787/agr_outlook-2018-en

[B136] Oh D. Marshall D. L. (1993). Antimicrobial activity of ethanol, glycerol monolaurate or lactic acid against *Listeria monocytogenes*. *Int. J. Food Microbiol.* 20 239–246. 10.1016/0168-1605(93)90168-G 8110601

[B137] Olaya Galán N. N. Ulloa Rubiano J. C. Velez Reyes F. A. Fernandez Duarte K. P. Salas Cárdenas S. P. Gutierrez Fernandez M. F. (2016). In vitro antiviral activity of *Lactobacillus casei* and *Bifidobacterium adolescentis* against rotavirus infection monitored by NSP4 protein production. *J. Appl. Microbiol.* 120 1041–1051. 10.1111/jam.13069 26801008

[B138] Olnood C. G. Beski S. S. M. Choct M. Iji P. A. (2015). Use of *Lactobacillus johnsonii* in broilers challenged with *Salmonella sofia*. *Anim. Nutr.* 1 203–212. 10.1016/j.aninu.2015.07.001 29767137 PMC5945932

[B139] Oude Elferink S. J. Krooneman J. Gottschal J. C. Spoelstra S. F. Faber F. Driehuis F. (2001). Anaerobic conversion of lactic acid to acetic acid and 1, 2-propanediol by *Lactobacillus buchneri*. *Appl. Environ. Microbiol.* 67 125–132. 10.1128/AEM.67.1.125-132.2001 11133436 PMC92530

[B140] Pandiyan P. Balaraman D. Thirunavukkarasu R. George E. G. J. Subaramaniyan K. Manikkam S. (2013). Probiotics in aquaculture. *Drug Invent. Today* 5 55–59. 10.1016/j.dit.2013.03.003

[B141] Park Y. H. Hamidon F. Rajangan C. Soh K. P. Gan C. Y. Lim T. S. (2016). Application of probiotics for the production of safe and high-quality poultry meat. *Korean J. Food Sci. Anim. Resour.* 36 567–576. 10.5851/kosfa.2016.36.5.567 27857531 PMC5112418

[B142] Peng W. X. Marchal J. L. M. van der Poel A. F. B. (2018). Strategies to prevent and reduce mycotoxins for compound feed manufacturing. *Anim. Feed Sci. Technol.* 237 129–153. 10.1016/j.anifeedsci.2018.01.017

[B143] Penha Filho R. A. C. Díaz S. J. A. Fernando F. S. Chang Y.-F. Andreatti Filho R. L. Junior A. B. (2015). Immunomodulatory activity and control of *Salmonella* Enteritidis colonization in the intestinal tract of chickens by *Lactobacillus* based probiotic. *Vet. Immunol. Immunopathol.* 167 64–69. 10.1016/j.vetimm.2015.06.006 26099807

[B144] Perez R. H. Zendo T. Sonomoto K. (2018). Circular and leaderless bacteriocins: biosynthesis, mode of action, applications, and prospects. *Front. Microbiol.* 9:2085. 10.3389/fmicb.2018.02085 30233551 PMC6131525

[B145] Pérez-Cano F. J. Dong H. Yaqoob P. (2010). Immunobiology in vitro immunomodulatory activity of *Lactobacillus fermentum* CECT5716 and *Lactobacillus salivarius* CECT5713: two probiotic strains isolated from human breast milk. *Immunobiology* 215 996–1004. 10.1016/j.imbio.2010.01.004 20219262

[B146] Piccart K. Vásquez A. Piepers S. De Vliegher S. Olofsson T. C. (2016). Short communication: lactic acid bacteria from the honeybee inhibit the in vitro growth of mastitis pathogens. *J. Dairy Sci.* 99 2940–2944. 10.3168/jds.2015-10208 26830735

[B147] Piotrowska M. (2014). The adsorption of ochratoxin a by *Lactobacillus* species. *Toxins* 6 2826–2839. 10.3390/toxins6092826 25247265 PMC4179162

[B148] Pizzolitto R. P. Salvano M. A. Dalcero A. M. (2012). Analysis of fumonisin B 1 removal by microorganisms in co-occurrence with aflatoxin B 1 and the nature of the binding process. *Int. J. Food Microbiol.* 156 214–221. 10.1016/j.ijfoodmicro.2012.03.024 22503712

[B149] Pluske J. R. Turpin D. L. Kim J. (2018). Gastrointestinal tract (gut) health in the young pig. *Anim. Nutr.* 4 187–196. 10.1016/j.aninu.2017.12.004 30140758 PMC6104527

[B150] Popova T. (2017). Effect of probiotics in poultry for improving meat quality. *Curr. Opin. Food Sci.* 14 72–77. 10.1016/j.cofs.2017.01.008 24570468

[B151] Rattanachaikunsopon P. Phumkhachorn P. (2010). Lactic acid bacteria: their antimicrobial compounds and their uses in food production. *Ann. Biol. Res.* 1 218–228.

[B152] Ricke S. C. (2003). Perspectives on the use of organic acids and short chain fatty acids as antimicrobials. *Poult. Sci.* 82 632–639. 10.1093/ps/82.4.632 12710485

[B153] Riera Romo M. Perez-Martinez D. Castillo Ferrer C. (2016). Innate immunity in vertebrates: an overview. *Immunology* 148 125–139. 10.1111/imm.12597 26878338 PMC4863567

[B154] Rihakova J. Cappelier J.-M. Hue I. Demnerova K. Fédérighi M. Prévost H. (2010). In vivo activities of recombinant divercin V41 and its structural variants against *Listeria monocytogenes*. *Antimicrob. Agents Chemother.* 54 563–564. 10.1128/AAC.00765-09 19841145 PMC2798489

[B155] Rishi P. Preet Singh A. Garg N. Rishi M. (2014). Evaluation of nisin–β-lactam antibiotics against clinical strains of *Salmonella enterica* serovar Typhi. *J. Antibiot.* 67 807–811. 10.1038/ja.2014.75 24961707

[B156] Rizzo A. Fiorentino M. Buommino E. Donnarumma G. Losacco A. Bevilacqua N. (2015). Lactobacillus crispatus mediates anti-inflammatory cytokine interleukin-10 induction in response to *Chlamydia trachomatis* infection in vitro. *Int. J. Med. Microbiol.* 305 815–827. 10.1016/j.ijmm.2015.07.005 26372530

[B157] Rocha T. S. Baptista A. A. S. Donato T. C. Milbradt E. L. Okamoto A. S. Rodrigues J. C. Z. (2012). Evaluation of in vitro and in vivo adhesion and immunomodulatory effect of *Lactobacillus* species strains isolated from chickens. *Poult. Sci.* 91 362–369. 10.3382/ps.2011-01803 22252349

[B158] Rodríguez J. M. Martínez M. I. Kok J. (2002). Pediocin PA-1, a wide-spectrum bacteriocin from lactic acid bacteria. *Crit. Rev. Food Sci. Nutr.* 42 91–121. 10.1080/10408690290825475 11934133

[B159] Rodriguez-Estrada U. Satoh S. Haga Y. Fushimi H. Sweetman J. (2013). Effects of inactivated *Enterococcus faecalis* and mannan oligosaccharide and their combination on growth, immunity, and disease protection in rainbow trout. *N. Am. J. Aquac.* 75 416–428. 10.1080/15222055.2013.799620

[B160] Roselli M. Pieper R. Rogel-Gaillard C. de Vries H. Bailey M. Smidt H. (2017). Immunomodulating effects of probiotics for microbiota modulation, gut health and disease resistance in pigs. *Anim. Feed Sci. Technol.* 233 104–119. 10.1016/j.anifeedsci.2017.07.011

[B161] Ruas-Madiedo P. Medrano M. Salazar N. De Los Reyes-Gavilán C. G. Pérez P. F. Abraham A. G. (2010). Exopolysaccharides produced by *Lactobacillus* and *Bifidobacterium* strains abrogate in vitro the cytotoxic effect of bacterial toxins on eukaryotic cells. *J. Appl. Microbiol.* 109 2079–2086. 10.1111/j.1365-2672.2010.04839.x 20846331

[B162] Russo P. Capozzi V. Arena M. P. Spadaccino G. Dueñas M. T. López P. (2014). Riboflavin-overproducing strains of *Lactobacillus fermentum* for riboflavin-enriched bread. *Appl. Microbiol. Biotechnol.* 98 3691–3700. 10.1007/s00253-013-5484-7 24413973

[B163] Russo P. Pia M. Fiocco D. Capozzi V. Drider D. Spano G. (2017). International journal of food microbiology *Lactobacillus plantarum* with broad antifungal activity: a promising approach to increase safety and shelf-life of cereal-based products. *Int. J. Food Microbiol.* 247 48–54. 10.1016/j.ijfoodmicro.2016.04.027 27240933

[B164] Sachsenrödder J. Twardziok S. O. Scheuch M. Johne R. (2014). The general composition of the faecal virome of pigs depends on age, but not on feeding with a probiotic bacterium. *PLoS One* 9:e88888. 10.1371/journal.pone.0088888 24586429 PMC3929612

[B165] Safwat A. M. Sarmiento-Franco L. Santos-Ricalde R. H. Nieves D. Sandoval-Castro C. A. (2015). Estimating apparent nutrient digestibility of diets containing *Leucaena leucocephala* or *Moringa oleifera* leaf meals for growing rabbits by two methods. *Asian Austral. J. Anim. Sci.* 28 1155–1162. 10.5713/ajas.14.0429 26104524 PMC4478484

[B166] Saint-Cyr M. J. Haddad N. Taminiau B. Poezevara T. Quesne S. Amelot M. (2017). Use of the potential probiotic strain *Lactobacillus salivarius* SMXD51 to control *Campylobacter jejuni* in broilers. *Int. J. Food Microbiol.* 247 9–17. 10.1016/j.ijfoodmicro.2016.07.003 27432696

[B167] Saleem A. M. Zanouny A. I. Singer A. M. (2017). Growth performance, nutrients digestibility, and blood metabolites of lambs fed diets supplemented with probiotics during pre- and post-weaning period. *Asian Austral. J. Anim. Sci.* 30 523–530. 10.5713/ajas.16.0691 28002935 PMC5394838

[B168] Santini C. Baffoni L. Gaggia F. Granata M. Gasbarri R. Di Gioia D. (2010). Characterization of probiotic strains: an application as feed additives in poultry against *Campylobacter jejuni*. *Int. J. Food Microbiol.* 141 S98–S108. 10.1016/j.ijfoodmicro.2010.03.039 20452074

[B169] Schlee M. Harder J. Köten B. Stange E. F. Wehkamp J. Fellermann K. (2008). Probiotic lactobacilli and VSL#3 induce enterocyte beta-defensin 2. *Clin. Exp. Immunol.* 151 528–535. 10.1111/j.1365-2249.2007.03587.x 18190603 PMC2276967

[B170] Schnürer J. Magnusson J. (2005). Antifungal lactic acid bacteria as biopreservatives. *Trends Food Sci. Technol.* 16 70–78. 10.1016/j.tifs.2004.02.014

[B171] Seal B. S. Drider D. Oakley B. B. Brüssow H. Bikard D. Rich J. O. (2018). Microbial-derived products as potential new antimicrobials. *Vet. Res.* 49:66. 10.1186/s13567-018-0563-5 30060765 PMC6066938

[B172] Seal B. S. Lillehoj H. S. Donovan D. M. Gay C. G. (2013). Alternatives to antibiotics: a symposium on the challenges and solutions for animal production. *Anim. Health Res. Rev.* 14 78–87. 10.1017/S1466252313000030 23702321

[B173] Seddik H. A. Bendali F. Gancel F. Fliss I. Spano G. Drider D. (2017). *Lactobacillus plantarum* and its probiotic and food potentialities. *Probiotics Antimicrob. Proteins* 9 111–122. 10.1007/s12602-017-9264-z 28271469

[B174] Seo J. K. Kim S. W. Kim M. H. Upadhaya S. D. Kam D. K. Ha J. K. (2010). Direct-fed microbials for ruminant animals. *Asian Austral. J. Anim. Sci.* 23 1657–1667. 10.5713/ajas.2010.r.08

[B175] Sha Y. Wang B. Liu M. Jiang K. Wang L. (2016a). Interaction between *Lactobacillus pentosus* HC-2 and *Vibrio parahaemolyticus* E1 in *Litopenaeus vannamei* in vivo and in vitro. *Aquaculture* 465 117–123. 10.1016/j.aquaculture.2016.09.007

[B176] Sha Y. Wang L. Liu M. Jiang K. Xin F. Wang B. (2016b). Effects of lactic acid bacteria and the corresponding supernatant on the survival, growth performance, immune response and disease resistance of *Litopenaeus vannamei*. *Aquaculture* 452 28–36. 10.1016/j.aquaculture.2015.10.014

[B177] Sharifuzzaman S. M. Austin B. (2017). “Probiotics for disease control in aquaculture,” in *Diagnosis and Control of Diseases of Fish and Shellfish*, eds Austin B. Newaj-Fyzul A. (Hoboken, NJ: John Wiley & Sons Ltd), 189–222. 10.1002/9781119152125.ch8

[B178] Shi S. H. Yang W. T. Yang G. L. Zhang X. K. Liu Y. Y. Zhang L. J. (2016). *Lactobacillus plantarum* vaccine vector expressing hemagglutinin provides protection against H9N2 challenge infection. *Virus Res.* 211 46–57. 10.1016/j.virusres.2015.09.005 26363195

[B179] Signorini M. L. Soto L. P. Zbrun M. V. Sequeira G. J. Rosmini M. R. Frizzo L. S. (2012). Research in veterinary science impact of probiotic administration on the health and fecal microbiota of young calves: a meta-analysis of randomized controlled trials of lactic acid bacteria. *Res. Vet. Sci.* 93 250–258. 10.1016/j.rvsc.2011.05.001 21620428

[B180] Singh A. P. Preet S. Rishi P. (2014). Nisin / beta-lactam adjunct therapy against *Salmonella enterica* serovar Typhimurium: a mechanistic approach. *J. Antimicrob. Chemother.* 69 1877–1887. 10.1093/jac/dku049 24633205

[B181] Singh V. P. (2018). Recent approaches in food bio-preservation-a review. *Open Vet. J.* 8 104–111. 10.4314/ovj.v8i1.16 29721439 PMC5918123

[B182] Sirichokchatchawan W. Temeeyasen G. Nilubol D. (2018). Protective effects of cell-free supernatant and live lactic acid bacteria isolated from thai pigs against a pandemic strain of porcine epidemic diarrhea virus. *Probiotics Antimicrob. Proteins* 10 383–390. 10.1007/s12602-017-9281-y 28434154 PMC7091344

[B183] Soto L. P. Zbrun M. V. Frizzo L. S. Signorini M. L. Sequeira G. J. Rosmini M. R. (2014). Effects of bacterial inoculants in milk on the performance of intensively reared calves. *Anim. Feed Sci. Technol.* 189 117–122. 10.1016/j.anifeedsci.2013.12.004

[B184] Soucy S. M. Huang J. Gogarten J. P. (2015). Horizontal gene transfer: building the web of life. *Nat. Rev. Genet.* 16 472–482. 10.1038/nrg3962 26184597

[B185] Souza Vera E. C. de Souza de Azevedo P. O. Domínguez J. M. Pinheiro and de Souza Oliveira R. (2018). Optimization of biosurfactant and bacteriocin-like inhibitory substance (BLIS) production by *Lactococcus lactis* CECT-4434 from agroindustrial waste. *Biochem. Eng. J.* 133 168–178. 10.1016/j.bej.2018.02.011

[B186] Speedy A. W. (2003). Global production and consumption of animal source foods. *J. Nutr.* 13 4048S–4053S. 10.1093/jn/133.11.4048S 14672310

[B187] Stern N. J. Eruslanov B. V. Pokhilenko V. D. Kovalev Y. N. Volodina L. L. Perelygin V. V. (2008). Bacteriocins reduce *Campylobacter jejuni* colonization while bacteria producing bacteriocins are ineffective. *Microb. Ecol. Health Dis.* 20 74–79. 10.1080/08910600802030196

[B188] Stevens M. Vollenweider S. Lacroix C. Zurich E. T. H. (2011). “The potential of reuterin produced by *Lactobacillus reuteri* as a broad spectrum preservative in food,” in *Protective Cultures, Antimicrobial Metabolites and Bacteriophages for Food and Beverage Biopreservation*, ed. Lacroix C. (New York, NY: Elsevier), 129–160. 10.1533/9780857090522.1.129

[B189] Sun K. Xie C. Xu D. Yang X. Tang J. Ji X. (2013). Lactobacillus isolates from healthy volunteers exert immunomodulatory effects on activated peripheral blood mononuclear cells. *J. Biomed. Res.* 27 116–126. 10.7555/JBR.27.20120074 23554802 PMC3602869

[B190] Surendran Nair M. Amalaradjou M. A. Venkitanarayanan K. (2017). *Antivirulence Properties of Probiotics in Combating Microbial Pathogenesis.* New York, NY: Elsevier Ltd. 10.1016/bs.aambs.2016.12.001 28189153

[B191] Szczurek W. Alloui M. N. Józefiak D. (2018). The effects of dietary whey lactose and *Lactobacillus agilis* bacteria on the growth performance, physicochemical conditions of the digestive tract and the caecal microbial ecology of broiler chickens. *Ann. Anim. Sci.* 18 483–500. 10.1515/aoas-2017-0045

[B192] Tannock G. W. Ghazally S. Walter J. Loach D. Brooks H. Cook G. (2005). Ecological behavior of *Lactobacillus reuteri* 100-23 is affected by mutation of the luxS gene. *Appl. Environ. Microbiol.* 71 8419–8425. 10.1128/AEM.71.12.8419 16332830 PMC1317450

[B193] Tellez G. Pixley C. Wolfenden R. E. Layton S. L. Hargis B. M. (2012). Probiotics/direct fed microbials for *Salmonella* control in poultry. *Food Res. Int.* 45 628–633. 10.1016/j.foodres.2011.03.047

[B194] Thammasorn T. Jitrakorn S. Charoonnart P. Sirimanakul S. Rattanarojpong T. Chaturongakul S. (2017). Probiotic bacteria (*Lactobacillus plantarum*) expressing specific double-stranded RNA and its potential for controlling shrimp viral and bacterial diseases. *Aquac. Int.* 25 1679–1692. 10.1007/s10499-017-0144-z

[B195] Tsai Y. T. Cheng P. C. Pan T. M. (2012). The immunomodulatory effects of lactic acid bacteria for improving immune functions and benefits. *Appl. Microbiol. Biotechnol.* 96 853–862. 10.1007/s00253-012-4407-3 23001058

[B196] Valeriano V. D. V. Balolong M. P. Kang D. K. (2016). Probiotic roles of *Lactobacillus* sp. in swine: insights from gut microbiota. *J. Appl. Microbiol.* 122 554–567. 10.1111/jam.13364 27914202

[B197] Veizaj-Delia E. Piu T. Lekaj P. Tafaj M. (2010). Using combined probiotic to improve growth performance of weaned piglets on extensive farm conditions. *Livest. Sci.* 134 249–251. 10.1016/j.livsci.2010.06.155

[B198] Vivier E. Zitvogel L. Lanier L. L. Yokoyama W. M. Ugolini S. (2011). Innate or adaptive immunity? The example of natural killer cells. *Science* 331 44–49. 10.1126/science.1198687 21212348 PMC3089969

[B199] Walter J. (2008). Ecological role of lactobacilli in the gastrointestinal tract: implications for fundamental and biomedical research. *Appl. Environ. Microbiol.* 74 4985–4996. 10.1128/AEM.00753-08 18539818 PMC2519286

[B200] Wang C. Chang T. Yang H. Cui M. (2015). Antibacterial mechanism of lactic acid on physiological and morphological properties of *Salmonella* Enteritidis, *Escherichia coli* and *Listeria monocytogenes*. *Food Control* 47 231–236. 10.1016/j.foodcont.2014.06.034

[B201] Wang J. Q. Yin F. G. Zhu C. Yu H. Niven S. J. De Lange C. F. M. (2012). Evaluation of probiotic bacteria for their effects on the growth performance and intestinal microbiota of newly-weaned pigs fed fermented high-moisture maize. *Livest. Sci.* 145 79–86. 10.1016/j.livsci.2011.12.024

[B202] Wang L. Liu C. Chen M. Ya T. Huang W. Gao P. (2015). A novel *Lactobacillus plantarum* strain P-8 activates beneficial immune response of broiler chickens. *Int. Immunopharmacol.* 29 901–907. 10.1016/j.intimp.2015.07.024 26481964

[B203] Wang H. Ni X. Qing X. Zeng D. Luo M. Liu L. (2017). Live probiotic *Lactobacillus johnsonii* BS15 promotes growth performance and lowers fat deposition by improving lipid metabolism, intestinal development, and gut microflora in broilers. *Front. Microbiol.* 8:1073. 10.3389/fmicb.2017.01073 28659893 PMC5466961

[B204] Wang X. Wang L. Huang X. Ma S. Yu M. Shi W. (2017). Oral delivery of probiotics expressing dendritic cell-targeting peptide fused with porcine epidemic diarrhea virus coe antigen: a promising vaccine strategy against PEDV. *Viruses* 9:312. 10.3390/v9110312 29068402 PMC5707519

[B205] Wang L. Li L. Lv Y. Chen Q. Feng J. Zhao X. (2018). *Lactobacillus plantarum* restores intestinal permeability disrupted by *Salmonella* infection in newly-hatched chicks. *Sci. Rep.* 8:2229. 10.1038/s41598-018-20752-z 29396554 PMC5797085

[B206] Wells J. M. (2011). Immunomodulatory mechanisms of lactobacilli. *Microb. Cell Fact.* 10(Suppl. 1):S17. 10.1186/1475-2859-10-S1-S17 21995674 PMC3231924

[B207] Wenk C. Pfirter H. P. Bickel H. (1980). Energetic aspects of feed conversion in growing pigs. *Livest. Prod. Sci.* 7 483–495. 10.1016/0301-6226(80)90086-X

[B208] Whittenbury R. (1964). Hydrogen peroxide formation and catalase activity in the lactic acid bacteria. *J. Gen. Microbiol.* 35 13–26. 10.1099/00221287-35-1-13 14167645

[B209] WHO (2015). *Global Action Plan on Antimicrobial Resistance.* Geneva: World Health Organization. 10.7196/samj.9644 26242647

[B210] Wong F. W. F. Ariff A. B. Abbasiliasi S. Stuckey D. C. (2017). Recovery of a bacteriocin-like inhibitory substance from *Pediococcus acidilactici* Kp10 using surfactant precipitation. *Food Chem.* 232 245–252. 10.1016/j.foodchem.2017.03.102 28490071

[B211] Woraprayote W. Malila Y. Sorapukdee S. Swetwiwathana A. Benjakul S. Visessanguan W. (2016). Bacteriocins from lactic acid bacteria and their applications in meat and meat products. *Meat Sci.* 120 118–132. 10.1016/j.meatsci.2016.04.004 27118166

[B212] World Bank (2017). *Drug-Resistant Infections: A Threat to Our Economic Future.* Available at: www.worldbank.org

[B213] Yang D. Chertov O. Bykovskaia S. N. Chen Q. Buffo M. J. Shogan J. (1999). beta-defensins: linking innate and adaptive immunity through dendritic and T cell CCR6. *Science* 286 525–528. 10.1126/science.286.5439.525 10521347

[B214] Yang E. Fan L. Jiang Y. Doucette C. Fillmore S. (2012). Antimicrobial activity of bacteriocin-producing lactic acid bacteria isolated from cheeses and yogurts. *AMB Express* 2:48. 10.1186/2191-0855-2-48 22963659 PMC3488010

[B215] Yang F. Hou C. Zeng X. Qiao S. (2015). The use of lactic acid bacteria as a probiotic in swine diets. *Pathogens* 4 34–45. 10.3390/pathogens4010034 25633489 PMC4384071

[B216] Yang W. Yang G. Shi S. Liu Y. Huang H. Jiang Y. (2017). Protection of chickens against H9N2 avian influenza virus challenge with recombinant *Lactobacillus plantarum* expressing conserved antigens. *Appl. Environ. Microbiol.* 101 4593–4603. 10.1007/s00253-017-8230-8 28353000

[B217] Yang Y. Galle S. Le M. H. A. Zijlstra R. T. Gänzle M. G. (2015). Feed fermentation with reuteran- and levan-producing *Lactobacillus reuteri* reduces colonization of weanling pigs by enterotoxigenic *Escherichia coli*. *Appl. Environ. Microbiol.* 81 5743–5752. 10.1128/AEM.01525-15 26070673 PMC4551235

[B218] Yang Y. Song H. Wang L. Dong W. Yang Z. Yuan P. (2017). Antiviral effects of a probiotic metabolic products against transmissible gastroenteritis coronavirus. *J. Probiotics Health* 5:184 10.4172/2329-8901.1000184

[B219] Yasuda K. Hashikawa S. Sakamoto H. Tomita Y. Shibata S. (2007). A new synbiotic consisting of *Lactobacillus casei* subsp. *casei* and dextran improves milk production in Holstein dairy cows. *J. Vet. Med. Sci.* 69 205–206. 10.1292/jvms.69.205 17339767

[B220] Yirga H. (2015). The use of probiotics in animal nutrition. *J. Probiotics Health* 3:132 10.4172/2329-8901.1000132

[B221] Yu L. Zhai Q. Tian F. Liu X. Wang G. Zhao J. (2016). Potential of *Lactobacillus plantarum* CCFM639 in protecting against aluminum toxicity mediated by intestinal barrier function and oxidative stress. *Nutrients* 8:783. 10.3390/nu8120783 27918411 PMC5188438

[B222] Yu L. Zhai Q. Zhu J. Zhang C. Li T. Liu X. (2017). Dietary *Lactobacillus plantarum* supplementation enhances growth performance and alleviates aluminum toxicity in tilapia. *Ecotoxicol. Environ. Saf.* 143 307–314. 10.1016/j.ecoenv.2017.05.023 28570951

[B223] Zhang C. N. Zhang J. L. Guan W. C. Zhang X. F. Guan S. H. Zeng Q. H. (2017). Effects of *Lactobacillus delbrueckii* on immune response, disease resistance against *Aeromonas hydrophila*, antioxidant capability and growth performance of *Cyprinus carpio* Huanghe var. *Fish Shellfish Immunol.* 68 84–91. 10.1016/j.fsi.2017.07.012 28698125

[B224] Zhao X. Zhen Z. Wang X. Guo N. (2017). Synergy of a combination of nisin and citric acid against *Staphylococcus aureus* and *Listeria monocytogenes*. *Food Addit. Contam. Part A* 34 2058–2068. 10.1080/19440049.2017.1366076 28795907

[B225] Zheng X. Duan Y. Dong H. Zhang J. (2017). Effects of dietary *Lactobacillus plantarum* in different treatments on growth performance and immune gene expression of white shrimp *Litopenaeus vannamei* under normal condition and stress of acute low salinity. *Fish Shellfish Immunol.* 62 195–201. 10.1016/j.fsi.2017.01.015 28108342

[B226] Zhitnitsky D. Rose J. Lewinson O. (2017). The highly synergistic, broad spectrum, antibacterial activity of organic acids and transition metals. *Sci. Rep.* 7:44554. 10.1038/srep44554 28294164 PMC5353632

[B227] Zhu Y. H. Li X. Q. Zhang W. Zhou D. Liu H. Y. Wang J. F. (2014). Dose-dependent effects of *Lactobacillus rhamnosus* on serum interleukin-17 production and intestinal T-cell responses in pigs challenged with *Escherichia coli*. *Appl. Environ. Microbiol.* 80 1787–1798. 10.1128/AEM.03668-13 24389928 PMC3957626

[B228] Zimmermann J. A. Fusari M. L. Rossler E. Blajman J. E. Romero-Scharpen A. Astesana D. M. (2016). Effects of probiotics in swines growth performance: a meta-analysis of randomised controlled trials. *Anim. Feed Sci. Technol.* 219 280–293. 10.1016/j.anifeedsci.2016.06.021

[B229] Zoghi A. Khosravi-Darani K. Sohrabvandi S. (2014). Surface binding of toxins and heavy metals by probiotics. *Mini Rev. Med. Chem.* 14 84–98. 10.2174/1389557513666131211105554 24329992

